# Circulating bacterial signature is linked to metabolic disease and shifts with metabolic alleviation after bariatric surgery

**DOI:** 10.1186/s13073-021-00919-6

**Published:** 2021-06-22

**Authors:** Rima M. Chakaroun, Lucas Massier, Anna Heintz-Buschart, Nedal Said, Joerg Fallmann, Alyce Crane, Tatjana Schütz, Arne Dietrich, Matthias Blüher, Michael Stumvoll, Niculina Musat, Peter Kovacs

**Affiliations:** 1 Medical Department III – Endocrinology, Nephrology, Rheumatology, University of Leipzig Medical Center, Leipzig, Germany; 2 Department of Medicine (H7), Karolinska Institutet, C2-94, Karolinska University Hospital, Stockholm, Sweden; 3 German Centre for Integrative Biodiversity Research (iDiv) Halle-Jena-Leipzig, Leipzig, Germany; 4 Helmholtz Centre for Environmental Research GmbH – UFZ, Halle, Germany; 5 Department of Isotope Biogeochemistry, Helmholtz Centre for Environmental Research – UFZ, Leipzig, Germany; 6 Department of Computer Science and Interdisciplinary Center for Bioinformatics, University of Leipzig, Leipzig, Germany; 7 Department of Visceral, Transplantation, Thoracic and Vascular Surgery, Section of Bariatric Surgery, Leipzig University Hospital, Leipzig, Germany; 8 Helmholtz Institute for Metabolic, Obesity and Vascular Research (HI-MAG) of the Helmholtz Zentrum München at the University of Leipzig and University Hospital Leipzig, Leipzig, Germany; 9 Deutsches Zentrum für Diabetesforschung, Neuherberg, Germany

**Keywords:** Blood bacteria, Obesity, Metabolic syndrome, Type 2 diabetes, Bariatric surgery

## Abstract

**Background:**

The microbiome has emerged as an environmental factor contributing to obesity and type 2 diabetes (T2D). Increasing evidence suggests links between circulating bacterial components (i.e., bacterial DNA), cardiometabolic disease, and blunted response to metabolic interventions. In this aspect, thorough next-generation sequencing-based and contaminant-aware approaches are lacking. To address this, we tested whether bacterial DNA could be amplified in the blood of subjects with obesity and high metabolic risk under strict experimental and analytical control and whether a putative bacterial signature is related to metabolic improvement after bariatric surgery.

**Methods:**

Subjects undergoing bariatric surgery were recruited into sex- and BMI-matched subgroups with (n = 24) or without T2D (n = 24). Bacterial DNA in the blood was quantified and prokaryotic 16S rRNA gene amplicons were sequenced. A contaminant-aware approach was applied to derive a compositional microbial signature from bacterial sequences in all subjects at baseline and at 3 and 12 months after surgery. We modeled associations between bacterial load and composition with host metabolic and anthropometric markers. We further tested whether compositional shifts were related to weight loss response and T2D remission. Lastly, bacteria were visualized in blood samples using catalyzed reporter deposition (CARD)-fluorescence in situ hybridization (FISH).

**Results:**

The contaminant-aware blood bacterial signature was associated with metabolic health. Based on bacterial phyla and genera detected in the blood samples, a metabolic syndrome classification index score was derived and shown to robustly classify subjects along their actual clinical group. T2D was characterized by decreased bacterial richness and loss of genera associated with improved metabolic health. Weight loss and metabolic improvement following bariatric surgery were associated with an early and stable increase of these genera in parallel with improvements in key cardiometabolic risk parameters. CARD-FISH allowed the detection of living bacteria in blood samples in obesity.

**Conclusions:**

We show that the circulating bacterial signature reflects metabolic disease and its improvement after bariatric surgery. Our work provides contaminant-aware evidence for the presence of living bacteria in the blood and suggests a putative crosstalk between components of the blood and metabolism in metabolic health regulation.

**Supplementary Information:**

The online version contains supplementary material available at 10.1186/s13073-021-00919-6.

## Background

The major health burden of obesity is largely attributable to its associated diseases such as type 2 diabetes (T2D), the metabolic syndrome (MetS), and cardiovascular diseases. Along known risk factors, such as genetic predisposition, poor diet, and lower physical activity, shifts in the gut microbial composition have been shown to contribute to metabolic inflammation at the advent of obesity and T2D [1–5]. Beyond composition, low gut bacterial diversity has been associated with increased obesity, insulin resistance, dyslipidemia, and increased inflammation [6, 7]. More recently, low gut bacterial load has been identified as a key driver related to chronic inflammatory states such as Crohn’s disease [8]. Converging evidence further points to an important role of the gut microbiome as a therapeutic target and prognostic marker: Weight loss interventions, such as diet and bariatric surgery, profoundly affect the gut microbiota composition, leading to an increased bacterial gene count and bacterial richness. This, in turn, is associated with a decrease in inflammatory markers and an increase in insulin sensitivity up to complete remission of T2D [9–11]. Accordingly, weight loss interventions are less effective in improving insulin resistance and inflammatory state in patients with low bacterial gene diversity [7].

Although most evidence has been submitted for the gut microbiome, host tissues—including the blood [12], liver, and adipose tissue [13, 14]—have been shown to accommodate microbial consortia finally accessible through culture-independent techniques. Few studies further linked increased bacterial DNA load in the circulation with the incidence of T2D [15, 16] and cardiovascular disease [17]. Moreover, bacterial signature in the blood has been linked to the circulatory compartment it derives from (i.e., systemic vs portal circulation) [18], systemic inflammation [18], T2D presence [13, 14, 19], and metabolic disease severity [20]. Similarly, there is mounting evidence for the diagnostic application of circulating bacterial DNA highlighted in a recent work employing contaminant-aware approaches to show that it can discriminate between multiple types of cancer and is highly dependent on disease severity [21]. On the other hand, only one study contemplated the interplay between metabolic interventions and circulating bacterial markers: Subjects with an established bacterial DNA translocation based on qPCR detection did not experience a remission of T2D or significant improvement in insulin sensitivity despite significant weight loss after bariatric surgery [22]. These results advocate a closer look at blood-borne bacterial DNA, bacterial signatures and their role in health and disease.

It is worth noting that studies on bacterial load and composition in low bacterial biomass environments such as the blood are subject to technical biases including highly underestimated environmental contamination [23]. To our knowledge, only few studies have actively controlled for contamination so far [13, 14, 21], and only two included a bioinformatic contaminant-aware approach [13, 14]. We therefore applied a contamination-aware approach to test the hypothesis that the presence, composition, and load of bacterial DNA in the blood reflect obesity, metabolic risk factors, and inflammatory burden as well as weight loss associated changes in anthropometric and metabolic parameters after bariatric surgery.

## Methods

### Characterization of study participants and sample collection

Sixty-four subjects were recruited at the University of Leipzig Medical Center, Germany, after matching for BMI and sex differing on T2D status with 32 subjects having no T2D and 32 with T2D according to ADA criteria [24]. However, 16 subjects had to be excluded from longitudinal comparisons due to missing follow-up samples in at least one timepoint (n = 15) or missing follow-up phenotypes (n = 1). If not indicated otherwise, 48 subjects (24 with T2D, 24 without) were included in all analyses (Table 1—baseline cohort characteristics of subjects with complete follow-up (n = 48), Additional file 1: table S1—baseline cohort characteristics with initial matching (n = 64)). Individuals fulfilled the following inclusion criteria: (1) eligibility to bariatric surgery according to international clinical guidelines (BMI ≥ 40 kg/m^2^, or ≥ 35 kg/m^2^ with at least one obesity-associated metabolic disease) and internal clinical multidisciplinary panel, (2) no chronic or acute inflammatory disease as determined by blood cell counts and CRP or clinical signs of infection, (3) no evidence of coronary or peripheral artery disease, (4) no known thyroid dysfunction, (5) no antibiotics intake in the 3 months prior to the study visit, (6) no pregnancy or nursing, and (7) no NSAID intake within 78 h prior to the study visit.

**Table 1:** Baseline cohort characteristics of subjects with complete follow-up

Baseline characteristics	NGT	T2D	P-value
**N**	24	24	
**General**			
**Sex (F/M)**	18/6	17/7	1
**Age (years)**	46.8 ± 8.59	50.1 ± 7.94	**0.007**
**BMI (kg/m**^**2**^**)**	50.8 ± 6.59	49.0 ± 6.68	0.943
**WHR**	0.91 [0.87; 0.95]	0.98 [0.99; 1.03]	**0.006**
**GFR (ml/min/1.73m**^**2**^**)**	93.8 [80.6; 114]	94.0 [79.6; 110]	0.996
**Smoking status**			
**Active smokers, N (%)**	2 (8.70%)	3 (12.5%)	1
**Glycemia, insulin resistance, and antidiabetic medication intake**			
**HbA1c (%)**	5.38 [5.29; 5.58]	6.56 [5.95; 8.22]	**< 0.001**
**FPG (mmol/l)**	5.25 [4.98; 5.42]	7.42 [6.54; 11.7]	**< 0.001**
**HOMA-IR**	3.10 [2.27; 4.53]	8.59 [5.71; 13.8]	**< 0.001**
**Nr antidiabetics**	0.00 [0.00; 0.00]	1.00 [1.00; 3.00]	**< 0.001**
**Hypertension status and antihypertensive medication**			
**Hypertension, N (%)**	16 (66.7%)	22 (91.7%)	0.076
**Number of anti-HTN drugs, N**	1.00 [1.00; 3.00]	3.50 [1.00; 5.00]	0.067
**Systolic blood pressure (mmHg)**	131 ± 11.0	133 ± 14.7	0.665
**Diastolic blood pressure (mmHg)**	76.8 ± 8.26	76.4 ± 15.1	0.924
**Dyslipidemia and antihyperlipidemic medication intake**			
**LDL-C (mmol/l)**	3.25 ± 0.87	3.09 ± 0.93	0.545
**HDL-C (mmol/l)**	1.27 [1.02; 1.52]	1.10 [0.94; 1.30]	0.105
**TG (mmol/l)**	1.52 (0.69)	1.67 [1.42; 2.24]	**0.017**
**Statin, N (%)**	1 (4.17%)	7 (29.2%)	**0.048**
**Ezetimib, N (%)**	0.00 (0.00%)	2 (8.33%)	0.489
**Blood and inflammatory markers**			
**Hemoglobin (g/dl)**	13.4 [13.0; 14.5]	14.2 [13.4; 14.9]	0.115
**Leukocytes (Gpt/l)**	6.70 [5.25; 7.20]	8.30 [7.65; 10.7]	**< 0.001**
**CRP (pg/ml)**	10.4 [3.47; 17.9]	8.72 [5.24; 22.8]	0.546

Median and first, as well as third quartile limits (median [q1; q3]) are shown for non-normally distributed, continuous variables. For normally distributed, continuous variables, data are given in mean ± standard deviation (mean ± SD). For categorical parameters, total numbers (percentage) are shown. Significant p-values are depicted in bold. *Abbreviations*: *BMI* body mass index, *CRP* C-reactive protein, *FPG* fasting plasma glucose, *HbA1c* glycated hemoglobin A1c, *HDL* high-density lipoprotein, *HOMA-IR* homeostatic model assessment for insulin resistance, *LDL* low-density lipoprotein, *NGT* normal glucose tolerance, *T2D* type 2 diabetes, *TG* triglycerides, *WHR* waist to hip ratio

Subjects were extensively phenotyped before bariatric surgery and at month 3 and 12 post-bariatric surgery. Phenotyping included clinical and anthropometric assessment in conjunction with the evaluation of patient medical history and physical examination. Among other parameters, blood pressure, waist and hip circumference measurements, and bioimpedance measurement for body composition analysis, including automatic calculation of visceral fat rating (BC-418 MA, Tanita, Tokyo, Japan), were assessed. Obesity-associated diseases such as T2D [24], hypertension [25], and hyperlipidemia [26] were defined according to the medical association definition of the specific disease. Metabolic syndrome (MetS) was defined according to international conventions [27]. Excess BMI loss (EBL) was calculated as follows: EBL = ((BMI_baseline_ − BMI_12 months_)/BMI_baseline_−25) × 100 [28], and poor response was defined as an EBL < 50%, whereas a good response was defined as an EBL > 60% (adapted from [28]) (Additional file 1: table S2—phenotypes of good vs poor responders).

### Replication cohort

To validate results from bacterial quantification and associations thereof with host parameters, data from a subgroup consisting of 62 subjects belonging to a previously described cohort from the same center were analyzed (Additional file 1: Table S3—phenotype replication cohort). Phenotyping procedures and cohort description of the whole cohort are available elsewhere [14]. In brief, patients are part of a larger study including 75 subjects with obesity undergoing laparoscopic RYGB and were recruited at the University of Leipzig Medical Center, Germany. Exclusion criteria were the same as for the primary cohort. Subjects received clinical phenotyping consisting of oral glucose tolerance test, collection of anthropometric (age, sex, and body mass index (BMI)) and metabolic parameters such as fasting plasma glucose (FPG), fasting plasma insulin (FPI), high- and low-density lipoprotein (HDL, LDL) cholesterol, homeostasis model assessment for insulin resistance (HOMA-IR), and hemoglobin A1c (HbA1c). Moreover, blood cell counts and hsCRP were measured in local routine laboratory measurements. Tumor necrosis factor alpha (TNF-α) and interleukin 6 (IL-6) were measured by high-sensitive ELISA (R&D Systems, Minneapolis; HSTA00E and HS600B) according to the manufacturer’s protocol.

### Sample preparation

Blood samples were collected after an overnight fast at each timepoint, and EDTA blood samples were collected for DNA isolation and stored at −20 °C. FPG was measured using the hexokinase method, HDL and LDL cholesterol were measured using enzymatic assays, and FPI was measured using chemiluminescence assay according to standard laboratory procedures. C-reactive protein (CRP) was measured using an Image Automatic Immunoassay System (Beckman Coulter). All measurements were performed according to routine laboratory procedures. HOMA-IR was calculated as previously described [29]. Lipopolysaccharide binding protein (LBP) was measured using the HK315 HUMAN LBP ELISA Kit (Hycult Biotech, Uden, Netherlands) according to the manufacturer’s recommendations. Samples and follow-up availability dictated the sample size used for analyses at each step as illustrated in Additional file 1: Fig. S1.

### Bacterial DNA extraction, quantification, and amplification for sequencing

#### Bacterial DNA extraction

To minimize contamination, all steps were performed under a sterile class II laminar flow hood using aseptic measures including the use of one-way lab coats, elbow-length gloves, and facemasks. All non-organic liquids and required materials were subjected to at least 120 min of UV radiation. Furthermore, negative controls consisting of UV-treated PBS (filled in EDTA vials using venipuncture ware from the same batch to simulate venous puncture) were included and carried through all experimental procedures, including sequencing.

DNA was extracted from 200 μl whole EDTA blood using the QIAMP Blood MiniKit (Qiagen, Germany) following the manufacturer’s recommendations with an additional lysozyme step at 37 °C overnight (0.25 mg/ml, L7615, Sigma-Aldrich, MO, USA). DNA yield, integrity, and quality were assessed using Quant-iT PicoGreen dsDNA kit (Invitrogen, CA, USA) and Nanodrop 2000 spectrometer (Thermo Fisher Scientific, MA, USA).

#### Quantification of bacterial 16S rRNA gene

Bacterial DNA was quantified by qPCR amplification of the bacterial 16S rRNA gene using previously published primers (F_Bact 1369: 5′-CGGTGAATACGTTCCCGG-3′ and R_Prok 1492:5′-TACGGCTACCTTGTTACGACTT-3′) [17, 30, 31]. All qPCR reactions were performed in duplicate each, using 50 ng of whole extracted DNA, prepared in a PCR clean room, and run on a LightCycler 480 (Hoffmann-La Roche, Basel, Switzerland) with the following conditions: 50 °C for 2 min, 95 °C for 10 min and 40 cycles of denaturation at 95 °C for 15 s, annealing at 60 °C for 30 s, and extension at 72 °C for 30 s. The amount of amplified bacterial DNA was calculated using mean Cp values for each duplicate against a standard curve from *E. coli* JM 109 DNA dilutions (Promega, MA, USA), which included seven duplicates ranging from 1.25 fg to 0.2 ng total bacterial DNA. Quantification of the analyzed samples was performed in three runs with R^2^ ≥ 0.995 and a mean PCR efficiency of 2.06 ± 0.03. Analysis was conducted according to the Livak method [32]. Results were in concordance with a commercially available kit (Zymoresearch, CA, USA) (n = 13, r = 0.74, p = 0.004) and proved more sensitive (ΔCt = 3.9). Obtained concentrations were normalized to the total concentration of extracted DNA as well as the used blood volume and are given as pg bacterial DNA per μg of isolated DNA. This is due to the variation of amounts of total extracted DNA from the same 200 μl blood volume for each sample, which ranged from 12.7 to 98.2 ng/μl. Blood bacterial quantity in the replication cohort was independently measured according to the same protocol. To overcome inter-assay variability, blood bacterial load was standardized (z-score transformation) within the cohorts and analyses were conducted on standardized bacterial quantities for each cohort separately as well as both of them combined.

#### 16S rRNA gene sequencing analysis

After extensive testing of combinations of primers and polymerases for amplification of prokaryotic/bacterial 16S rRNA variable regions [14], V4-V5 primers adapted from the Ribosomal Database Project (RDP) (V4-F: 5′-ACTGGGCGTAAAGCG-3′; V5-R: 5′-CCGTCAATTCCTTTGAGTTT-3′) were used [33]. PCR reactions were prepared in triplicate in a sterile laminar flow hood and performed using 50 ng total DNA in a total reaction volume of 25 μl containing 1.25 μl of each primer (10 μM), 12.5 μl 2x Q5 Reaction Buffer (including Q5® High-Fidelity DNA Polymerase, New England BioLabs, MA, USA), and 6.25 μl UV-ed PCR-grade water. The reaction was carried out on a LightCycler 480 (Hoffmann-La Roche, Basel, Switzerland) under the following conditions: 98 °C for 2 min and 40 cycles of denaturation at 98 °C for 30 s, annealing at 58 °C for 30 s, and extension at 72 °C for 30 s. Each PCR reaction included at least 3 negative controls in addition to the extraction controls (blank controls) and the absence of detectable PCR products in these negative controls was confirmed by gel electrophoresis. Regardless of this, negative controls for each run were pooled and carried through sequencing to be used for contaminant identification. Replicate amplicons were pooled and purified using Agencourt Ampure magnetic purification beads (Beckman Coulter Indianapolis, IN, USA) according to the manufacturer’s protocol to remove short amplification products and primer dimers. DNA amounts were quantified using Quant-iT PicoGreen dsDNA kit (Invitrogen, CA, USA). Library preparation and paired-end sequencing were performed commercially (BGI Genomic, Shenzhen, China) on Illumina Hiseq2500 technology and using custom fusion primers in a one-step PCR approach.

### Bioinformatics and statistical analyses

#### Statistical analyses

Statistical analyses were performed in R v3.5.0 (R Development Core Team, 2008). Prior to statistical testing, distribution and single test assumptions were checked to ensure the test's suitability. For cross-sectional comparisons between two groups, the Wilcoxon signed-rank test was used for non-normally distributed variables according to Shapiro-Wilk testing, whereas unpaired Student’s t-test was used to compare normally distributed variables. Median and first as well as third quartile limits are depicted for non-normally distributed variables, whereas for normally distributed continuous variables, data are given as mean ± standard deviation (SD). The Friedman test was used to compare dependent groups at different timepoints. Categorical parameters were analyzed using the chi-square test. Bivariate correlation analyses were performed using Spearman’s rank correlation test. Figures were generated using ggplot2 [34] v3.1.0, ggpubr v0.2 [35], and corrplot v.0.84 [36] as well as phyloseq v1.26.1 [37] and relabeled in Adobe Illustrator (Adobe Inc., CA, USA). A p-value threshold of 0.05 was used to depict statistical significance. Analyses were adjusted for multiple hypotheses *ad modum* Benjamini-Hochberg, in which case a false discovery rate (FDR) of *Padj* < 0.05 was considered significant.

#### Processing of 16S rRNA reads and amplicon sequence variant (ASV) clustering

A total of 12,857,649 Illumina Hiseq quality filtered paired-end reads were obtained from BGI, with samples having a median read count of 63,055 [9661–64,630] [38]. Preprocessing and quality filtering consisted of removing reads with a certain proportion of low-quality (20) bases (20% of read original length), contaminated by adapter (5 bases overlapped by reads and adapter with maximal 3 bases mismatch allowed), with ambiguous bases and with low complexity (reads with 10 consecutive same base). Subsequently, quality was checked using Multiqc [39] and data was imported to QIIME2 [40] V2019.1 for analysis [38, 41]. Denoising, dereplication, merging, and chimera filtering as well as ASV inference were done in one step using the DADA2 [42] plugin in QIIME 2, resulting in 40269 ± 6033 non-chimeric reads with an average read length of 329 bp. Reads were truncated at a length of 242 bp. For phylogenetic analyses, primer-truncated and quality trimmed reads were used to create an alignment using mafft [43]. The alignment was masked to remove uninformative highly variable regions and a rooted tree was generated using the align-to-tree-mafft-fasttree plugin. For taxonomic classification, a scikit-learn [44] naive Bayes classifier was created against the taxonomic classification from ARB-SILVA [45] 132 release (99% OTU data set), which was trained for the used primers. Only reads mapped to bacterial taxonomy were retained. This resulted in a total of 2860 bacterial ASVs with a total frequency of 7,784,509. Derived taxonomy, tree, and feature table were imported into phyloseq [37] v1.26.1, and all subsequent analyses were performed in R v 3.5.0 0 (R Development Core Team, 2008).

#### Bacterial contaminant identification in blood samples

A bioinformatic decontamination step using the Decontam [46] package v1.2.1, which is intended for the identification of contaminants in low bacterial biomass samples, was added. To identify contaminant ASVs, the “prevalence” method was applied. A binomial distribution with low scores for low prevalence ASVs was found around 0.1 and high prevalence ASVs with higher confidence increasing around 0.175, which led us to select a data-driven contaminant score of 0.175 instead of the default classification score 0.1 (Additional file 1: Fig. S2). Using these parameters, 114 features were classified as potential contaminants and removed, leading to a total of 2746 ASVs (Additional file 2: Table S2, Additional file 2: Table S3, Additional file 2: Table S4). The pruned phyloseq object was then used for subsequent analyses. We moreover added a more stringent decontamination step using a contaminant score of 0.5 leading to flagging of each of the 172 ASVs appearing in the negative samples as contaminants. While ASVs derived from both decontamination scores led to similar results in associations of taxonomy with host variables (data not shown), the data-driven decontamination at a lower score was more likely to influence differential abundance analyses. This led us to use a more stringently decontaminated taxonomy while performing differential abundance testing to avoid spurious observations. The stringently decontaminated taxonomy included a total of 2688 ASVs (Additional file 2: Table S5, Additional file 2: Table S6).

#### Analyses of bacterial composition and its association with host phenotypes

Alpha diversity measures (Shannon diversity, observed richness) were calculated and RDA on Bray-Curtis-dissimilarity distances was conducted to identify host covariates contributing to bacterial community composition in the blood in vegan [47] v2.5-4. Samples with missing observations (NAs) in the covariates were eliminated from the dataset prior to analyses. Variables used in the RDA model included T2D status, sex, metformin use, PPI use, timepoint, MetS status, diabetes alleviation after surgery, EBL, surgical procedure, BMI, systolic blood pressure, eGFR, CRP, HbA1c, HDL cholesterol, LDL cholesterol, triglycerides, total fat mass in percentage, visceral fat ratio, number of antidiabetics, number of antihyperlipidemic drugs, number of antihypertensive drugs, white blood cell count, age, bacterial load, and waist to hip ratio. We further used automatic stepwise modeling and model selection using the ordistep approach both ways with 999 permutations to identify the most significant covariates contributing to community composition. Microbiome [48] v1.4.2. was used to perform correlation analyses between relative taxa abundances and host variables of interest applying Spearman’s rank correlation.

A random forest classification (randomForest [49] v4.6-14) approach on both phylum- and genus-level abundances was used to develop classification indices for MetS for subjects at baseline. For phylum- and genus-level, NGS -derived read counts from MetS and non-MetS subjects were normalized by the total read count of the corresponding phylum or genus and used as features for training of a random forest classifier. The classification index predicted ranges between 0 and 1 and corresponds to the out-of-bag predicted probability of being classified as belonging to the class of interest (i.e., having MetS) with higher scores indicating higher probability for having MetS. Performance of classification indices was quantified using receiver operating characteristic curve and AUC using pROC [50] v1.14.0. Cross validation was performed using the rfUtilities package [51, 52] v2.1-5. Specifics of random forest classification are as follows: number of trees 500; number of variables tried at each split 11; OOB estimate of error rate at genus level 21.3%, at phylum level 29.8%; classification accuracy for model at genus level: user accuracy 100, producer accuracy 79.1, model kappa = 0.147, model OOB error = 0.204, model error variance = 4.7 × 10^−5^; and classification accuracy for model at phylum level: user accuracy 91.2, producer accuracy 79.5, model kappa = 0.1357, model OOB error 0.25, model error variance = 7.7 × 10^−4^.

Differential abundance analyses for preassigned dichotomous groups were performed using DESeq2 [53] v1.22.2. Size factors for count data were calculated using the poscounts estimator. Dispersion estimates were performed using the DESeq command, a local regression model was used to fit and test the data, and the negative binomial Wald test was used to test differential abundance (log2 fold change) and significance. Results are reported for differentially abundant taxa with a significance *Padj* < 0.01 after multiple hypotheses correction *ad modum* Benjamini-Hochberg. Specifically, for the comparison between T2D remission and no remission following bariatric surgery, only subjects with T2D at baseline were retained in the analyses (Additional file 1: Fig. S3).

### Catalyzed reporter deposition (CARD)-fluorescence in situ hybridization (FISH)

#### Blood sample collection and cell separation

To visualize and quantify bacteria in the blood, samples from one patient post-bariatric surgery (male, 38 years, currently 60 months post-bariatric surgery, current BMI 30 kg/m^2^, no T2D, no MetS, initial BMI 61.9 kg/m^2^, EBL 54.5%) and a healthy control (male, 50 years, BMI 24.1 61.9 kg/m^2^, no known diseases, no medication) were collected before and 60 min after mixed meal intake on EDTA 1.6 mg/ml to avoid coagulation and kept overnight at 4°C in the fridge. Density gradient centrifugation was performed in an initial step to separate microbial cells from blood cells. Thus, 5 ml of 80% Nycodenz solution was added at the bottom of a tube containing 5 ml of blood, followed by centrifugation at 13000 rpm, 4°C for 2 h. After density gradient centrifugation, individual layers consisting of Supernatant and a Nycodenz layer could be separated. Each layer was individually collected and subjected to serial filtration through two consecutive 3 μm and one 0.22 μm pore size polycarbonate filters (GTTP type, 0.2 μm pore size PC membrane, 25 mm diameter, Merck Millipore, Germany). After filtration, all filters were washed in 1× UV-ed PBS buffer and were immersed in 4% paraformaldehyde solution (PFA) in 1× UV-ed PBS buffer for 2 h at room temperature. Following chemical fixation, filters were washed in 1× UV-ed PBS buffer, dehydrated in 80% ethanol, air dried, and stored at 4°C for CARD-FISH procedure.

*CARD-FISH* was performed following the standard protocol [54] with slight modifications. Permeabilization in Lysozyme solution (Sigma-Aldrich, MS, USA) (10 μg ml^−1^) in 0.1 M Tris-HCl (pH = 7.8) and 0.05 M EDTA (pH = 8) buffer was done for 30 min at 37°C followed by 0.01 M HCL for 10 min at room temperature (RT) with washing steps of 1 min at RT in ultrapure water in between treatments. Hybridization of filter pieces belonging to both 3 μm and 0.2 μm filters took place for 3 h at 46 °C in a pre-warmed hybridization buffer containing 0.9 M NaCl, 20 mM Tris-HCl (pH = 7.5), 10% (w/v) dextran sulfate, 0.02% (w/v) SDS, 35% (v/v) formamide (Fluka, Waltham, USA), and 1% (w/v) blocking reagent (Boehringer, Mannheim, Germany). The HRP-labeled probe applied is specific for bacteria (EUB 338, [55]) and was used at a concentration of 0.166 ng ml^− 1^ (HRP-probe stock solution of 50 ng ml^− 1^ diluted 1:300 v/v in hybridization buffer). Following hybridization, filter pieces were incubated in 50 ml of pre-warmed washing buffer containing 70 mM NaCl, 5 mM EDTA (pH = 8.0), 20 mM Tris-HCl (pH = 7.5), and 0.01% SDS for 15 min at 48 °C. After washing, filters were incubated for 15 min at RT in 1× PBS (pH = 7.6) to equilibrate the HRP-labeled probe. Subsequently, tyramide deposition was performed by incubating the filters for 20 min at 46 °C in the dark in amplification buffer containing 1× PBS, 2 M NaCl, 0.1% (w/v) blocking reagent, 10% (w/v) dextran sulfate, 0.0015% (v/v) H_2_O_2_, and 1 μg ml^−1^ Alexa 594-labeled tyramides (ThermoFisherScientific, MA, USA). Afterwards, the hybridized filters were rinsed in 1× PBS for 10 min at RT followed by staining with 4′,6-diamidino-2-phenylindole (DAPI) 1 μg ml^−1^ for 10 min at RT, washing in ultrapure water, air dried, and embedded in mounting medium (a mixture of low fluorescence glycerol mountant (Citifluor AF1, London) and mounting fluid Vecta Shield (Vecta Laboratories, CA, USA)) in a 4:1 v/v ratio and stored at −20°C prior to imaging.

#### Microscopic investigation

Approximately 20–30 randomly selected fields of view (each of 15130.8 μm^2^) were imaged on each hybridized filter piece from 3 μm and 0.2 μm pore size hybridized filters. The microscopic evaluation of the hybridized filters was done using a fluorescence microscope (Imager. Z2, Zeiss, Germany) with × 20 air (numerical aperture (NA) = 0.5) and × 63 oil (NA = 1.4) objectives. On the 3 μm filters, we observed predominantly unhybridized blood cells of different sizes for both Supernatant and Nycodenz filtrated samples, while hybridized bacterial cells were constantly found on the 0.22 μm filters of the Nycodenz filtrated samples, occasionally in very low abundances also on the 0.22 μm filters of the Supernatant filtrated samples (data not shown). Bacterial counts were performed only on the 0.22 μm filters from the Nycodenz filtrate layer on 20 to 22 randomly selected fields of view.

## Results

Our aim was to interrogate putative bacterial signatures related to obesity and characterize their potential dynamics after weight loss and metabolic alleviation following bariatric surgery (complete flowchart approach available under Additional file 1: Fig. S1—full description under the “Methods” section). For this, we started by investigating the predominant signature and taxonomy in a cross-sectional approach in blood samples from subjects at baseline (Table 1—baseline cohort characteristics of subjects with complete follow-up (n = 48), Additional file 1: table S1—baseline cohort characteristics with initial matching (n = 64)) and went on to further explore the change in taxonomy in blood samples available for all subjects with follow-up (Tables 1 and 3) at months 3 and 12 after the procedure. To explore bacterial signatures related to weight loss and diabetes alleviation, similar analyses were done in good vs poor responders (Additional file 1: table S2—phenotypes of good vs poor responders, Additional file 1: Fig. S3).

### Contaminant-aware analyses allow the derivation of a core blood bacterial signature

After filtering and removing contaminating ASVs (Additional file 2: Table S2), a distinct bacterial profile remained consisting of 20 phyla (Additional file 2: Table S4). Of the 2746 detected ASVs, 65 could not be assigned at the phylum level (2.4%). Assigned taxonomy was dominated by *Proteobacteria* (59.0%), *Firmicutes* (12.4%), *Patescibacteria* (11.97%), *Cyanobacteria* (8.9%), and *Actinobacteria* (2.3%), whereas 5.41% of ASVs belonged to various other phyla.

**Fig. 1:**
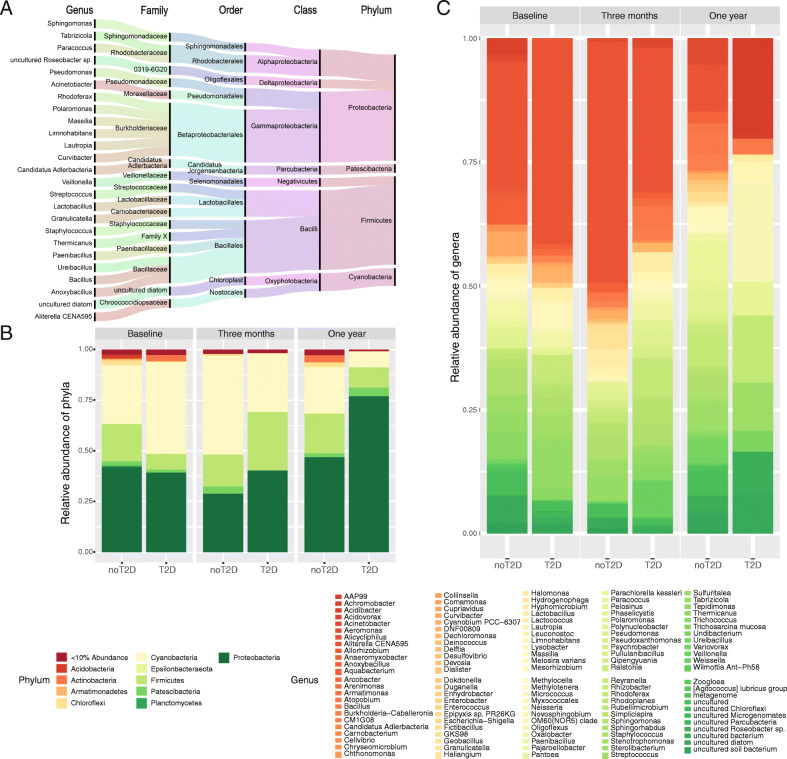
Contaminant-aware blood bacterial signatures at a decontam score of 0.175. **A** Top 25 genera and their respective taxonomic classification. The bandwidth is proportional to the amount of ASVs within each phylum, genera are ordered from bottom to top within each family according to alphabetical order, and taxonomy was generated with all available baseline samples (n = 64) and follow-up samples at 3 months (n = 48) and 1 year (n = 48). **B** Relative abundance of phyla within samples with follow-up sequencing at all timepoints: baseline (n = 48), 3 months (n = 48), and 1 year (n = 48) after bariatric surgery. Phyla with an abundance within the whole dataset of less than 10% are flagged under the category “< 10% Abundance.” Taxonomy is shown for matched subjects with and without T2D (n for each group = 24) at each timepoint separately. **C** Relative abundance of core genera belonging to the phyla with an abundance of > 10% within samples with follow-up sequencing at all timepoints: baseline (n = 48), 3 months (n = 48), and 1 year (n = 48) after bariatric surgery. Taxonomy is shown for matched subjects with and without T2D (n for each group = 24) at each timepoint separately

21.6% of all ASVs (592) could not be assigned at the genus level. Assigned ASVs belonged to 314 genera and the most abundant genera included *Aliterella, Anoxybacillus, Lactobacillus*, and *Sphingomonas* from the three dominant phyla (Fig. 1A). The core bacterial signature consisting of 10 phyla and 120 genera with an overall abundance of more than 10% in the dataset could be recovered at each timepoint, although quantitative changes could be tracked over time and within disease and response groups (see further results) (Fig. 1B, C).

### Bacterial community composition is influenced by medication and reflects metabolic health

Twenty-seven available host variables (Additional file 1: tables S4, S5) were fit onto genus-level RDA in all available samples with no missing data (n = 101). RDA showed that 39.7% of the observed variance could be explained by the collected host variables (R^2^ = 0.3969, R^2^adj = 0.162, p-values of Anova = 0.004). Associations with RDA ordination on Hellringer transformed abundances were most notable for timepoint (envfit; R^2^ = 0.2184, p = 0.001), white blood cell count (envfit; R^2^ = 0.2531, p = 0.001), BMI (envfit; R^2^ = 0.2219, p = 0.001), total fat mass (envfit; R^2^ = 0.2090, p = 0.001), bacterial quantity (envfit; R^2^ = 0.1788, p = 0.001), and triglycerides (envfit; R^2^ = 0.1560, p = 0.001). Several other significant associations with bacterial composition were observed for general host characteristics such as age, T2D status, MetS status, sex, anthropometric variables such as waist-to-hip ratio and visceral fat rating, and markers of metabolic control including HDL cholesterol and HbA1c as well as inflammation markers like CRP and bacterial DNA burden (Additional file 1: table S4: envfit output for 27 variables on genera level RDA, Additional file 1: Fig. S4). Similar results were observed at ASV levels as well (Additional file 1: table S5).

Interestingly, while the number of antihypertensive, antidiabetic, and antihyperlipidemic medication was not correlated with ordination, the use of metformin and PPI significantly contributed to variance in composition (metformin R^2^ = 0.0380, p = 0.022; PPI R^2^ = 0.0674, p = 0.001; Additional file 1: tables S4, S5).

### Blood bacterial signature allows robust classification of subjects with metabolic syndrome

**Fig. 2:**
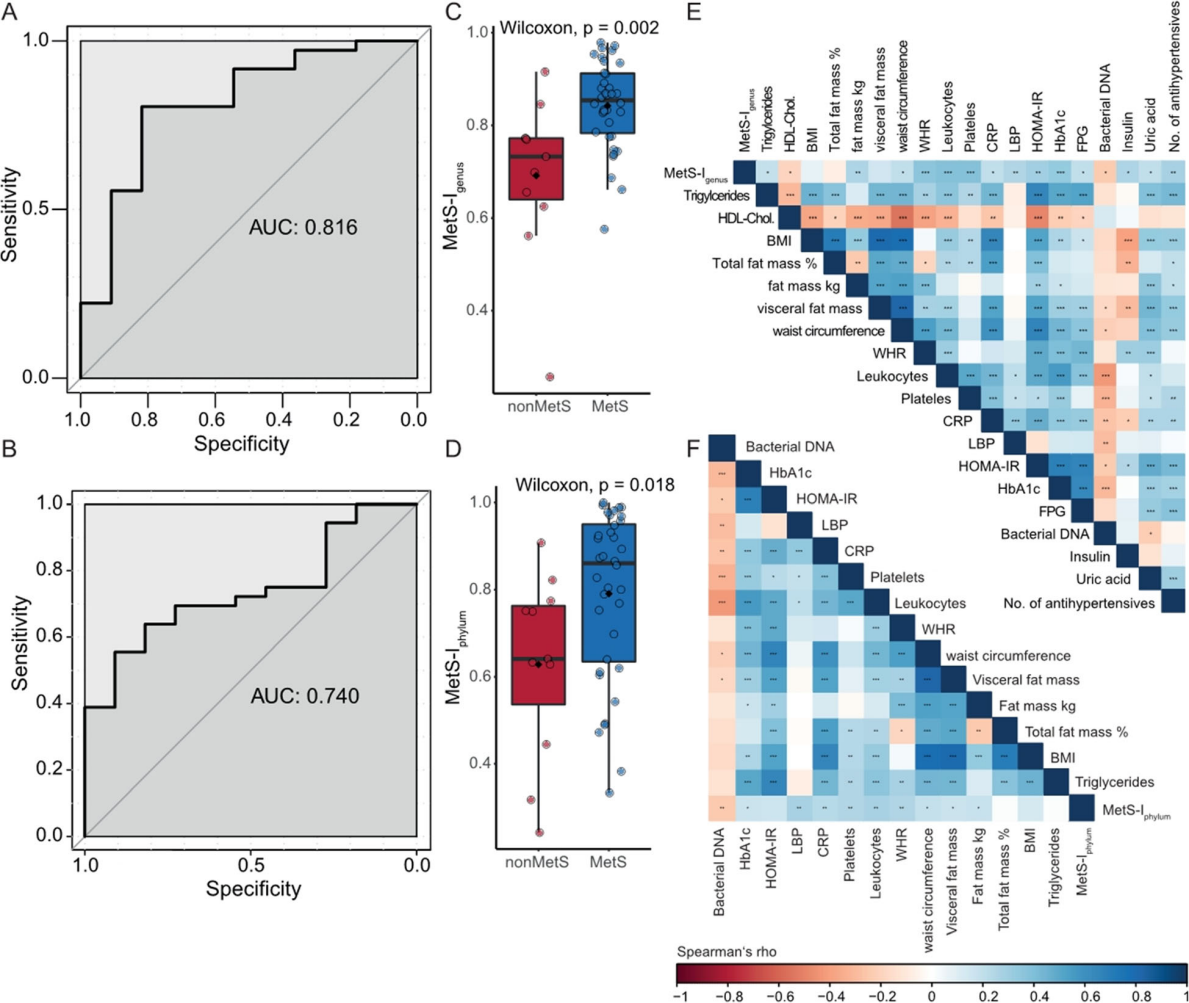
Bacterial blood signature derived metabolic syndrome classification index. Probability of being classified as a subject with metabolic syndrome using random forest classification of phyla and genera abundances (n phyla = 20, n genera = 314, in 47 subjects—36 with and 11 without MetS, missing n = 1, nondiabetic, not unambiguously categorized). **A**, **B** Corresponding area under the curve for both genus- and phylum-level random forest models for metabolic syndrome prediction respectively. **C**, **D** MetS-I_Genus_ and MetS-I_Phylum_ respectively in subjects with and without metabolic syndrome, boxplots with Tukey-whiskers and mean (◆) are shown. Unpaired samples Wilcoxon signed-rank test is used to compare groups and nominal p-values are shown. **E**, **F** Spearman’s rank correlations of MetS-I_Genus_ and MetS-I_Phylum_ at baseline with host variables from all timepoints. Nominal significant values are indicated: *p < 0.05, **p < 0.01, ***p < 0.001

Cardiovascular disease and mortality are increased in subjects with MetS [56] even in the absence of T2D. Because MetS status was significantly associated with bacterial composition beyond single risk factors used in the MetS definition [27] (Additional file 1: tables S4, S5), we sought to investigate if bacterial composition could predict MetS status. For this, we applied random forest classification on the genus- as well as phylum-level abundances (20 phyla and 314 genera) to develop a classification index. The resulting MetS classification index (MetS-I_genus_ and MetS-I_phylum_) could robustly classify subjects along their actual clinical group at baseline and performed better at genus than at phylum level (AUCgenus = 0.816, 95% CI 0.661–0.9705 (DeLong) and AUCphylum = 0.740 95% CI 0.589–0.890 (DeLong)) (Fig. 2A–D). MetS-I at both genus and phylum levels at baseline was correlated with several markers related to obesity, visceral fat distribution, insulin resistance, and inflammation from all timepoints, but was negatively associated with bacterial DNA load in the blood (Fig. 2E, F). The largest associations with host variables were seen for MetS-I_genus__,_ which was positively correlated with anthropometric markers related to obesity and insulin resistance such as total fat mass, waist circumference, and WHR. It was also positively associated with inflammation reflected in white blood cell count, CRP, and LBP as well as metabolic markers related to obesity and insulin resistance such as HOMA-IR, HbA1c, FPG, FPI, and uric acid. The MetS-I_genus_ was further related to dyslipidemia and hypertension with positive correlations noted for triglyceride levels and number of antihypertensive drugs, while being negatively associated with HDL cholesterol. Moreover, both MetS-I_genus_ and MetS-I_phylum_ were negatively associated with bacterial quantity (Fig. 2E, F, Table 2).

**Table 2:** Spearman correlations between MetS-I_genus_ at baseline with host variables (n = 141, 47 subjects at 3 timepoints)

Variables	Rho	p-value
CRP	0.21	< 0.05
Fasting glucose	0.2	< 0.05
Triglycerides	0.2	< 0.05
HDL cholesterol	−0.19	< 0.05
HbA1c	0.33	< 0.0001
Insulin	0.27	< 0.5
Total fat mass	0.26	< 0.01
Number of antihypertensive drugs	0.27	< 0.01
Platelet count	0.38	< 0.0001
Serum albumin	0.18	< 0.05
Uric acid	0.2	< 0.05
White blood cell count	0.38	< 0.0001
LBP	0.25	< 0.001
Bacterial quantity	−0.23	< 0.05

### T2D is characterized by a loss of health-associated genera and a decreased ASV richness

**Fig. 3:**
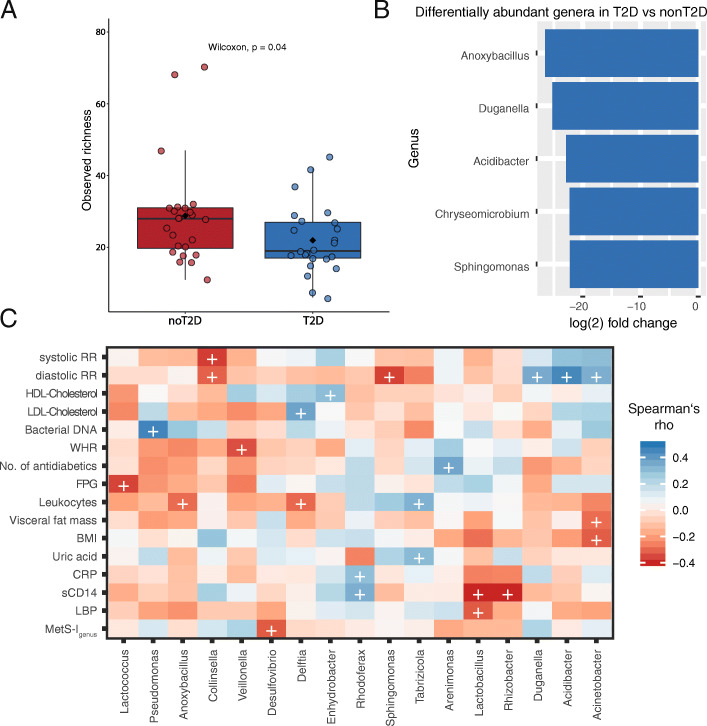
**A** Observed richness between subjects with (n = 24) and without T2D (n = 24), boxplots with Tukey-whiskers and mean (◆) as well as median are shown. Unpaired samples Wilcoxon signed-rank test is used to compare groups and nominal p-value is shown. **B** Differentially abundant genera in subjects with T2D (n = 24) compared to subjects without T2D (n = 24) at baseline. Differential abundance is calculated at ASV level using taxonomy after stringent control for contamination at a decontam score of 0.5 and reported at the genus level. **C** Spearman’s rank correlations of relative selected genera abundance with host parameters at baseline. Selection included genera seen to be differentially abundant between groups (i.e., T2D, good/poor responders, and T2D alleviation vs no T2D alleviation). Only genera are shown with at least one significant correlation with host markers. (+) refers to p-value < 0.05. Color represents correlation strength (Rho) according to color legend. *Abbreviations*: HDL high-density lipoprotein, LDL low-density lipoprotein, WHR waist to hip ratio, BMI body mass index, CRP C-reactive protein, sCD14 soluble cluster of differentiation 14, LBP lipopolysaccharide binding protein, EBL excess BMI loss

We investigated bacterial alpha diversity according to T2D status: At baseline, subjects with T2D (n = 24) displayed significantly lower observed richness as compared to subjects without T2D (n = 24) (mean observed richness = 28 ± 2.31 vs 19 ± 2.10, p = 0.039 in subjects without T2D and with T2D respectively; Fig. 3A). T2D was associated with significant shifts in several genera as compared to subjects without T2D: At baseline, significantly lower abundances of *Anoxybacillus*, *Duganella*, *Acidibacter*, and *Chryseomicrobium* as well as *Sphingomonas* are found in T2D (Fig. 3B). Furthermore, timepoint seemed to be relevant for the differences in bacterial abundances between subjects with and with T2D (Additional file 1: Fig. S5A, B). There were no significantly differentially abundant ASVs between T2D and nonT2D overlapping between baseline and 1 year, but the differences between subjects with and without T2D seemed to be shifting gradually. While subjects with T2D at baseline and 3 months showed a reduced abundance in *Sphingomonas*, a common feature seen between T2D at 3 months and 1 year was reduced *Pseudomonas* in T2D. Congruent T2D-associated features seen at baseline and 1 year were reduced *Bacillaceae* and *Burkholderiaceae* (Additional file 2: Table S7). Some of the reduced genera in T2D were expectedly negatively associated with markers of metabolic disease and inflammation such as *Anoxybacillus* and *Sphingomonas* at baseline with leukocytes and blood pressure respectively (Fig. 3C) as well as *Paracoccus* with HOMA-IR and leukocytes and *Rhodoferax* with markers of obesity at 1 year post-bariatric surgery (Additional file 1: Fig. S5C). Other genera were surprisingly positively associated with BMI such as *Sphingomonas* (at months 3 and 12) or with lipids and blood pressure such as *Acidibacter* (at baseline, months 3 and 12) (Fig. 3C, Additional file 1: Fig. S5C)

### Differential response in weight loss and glucose metabolism following bariatric surgery

As expected, bariatric surgery had a marked impact on BMI, body composition (WHR, total fat mass, and visceral fat ratio), metabolism, and inflammation as early as 3 months post-procedure (Table 3). From 24 subjects with T2D at baseline, nine subjects were still classified as T2D after 3 months, and only four after 1 year. From patients without T2D at 1 year, eight had impaired fasting glucose (prediabetes) and classified therefore as subjects without T2D alleviation after bariatric surgery. From the total 48 subjects with complete follow-up data, 24 subjects were categorized as good responders (i.e., losing more than 60% of excess BMI within 1 year), whereas 13 subjects were classified as poor responders (Additional file 1: table S2, Fig. S3). Poor responders not only lost less weight, but also benefited less from bariatric surgery in regard to improvement of lipid metabolism, insulin resistance, or inflammation, as they still displayed significantly higher HOMA-IR, triglycerides, LDL cholesterol, and CRP compared to good responders 1 year after bariatric surgery (Additional file 1: table S2).

**Table 3:** Clinical and biological characteristics of study cohort subjects before, 3, and 12 months after the bariatric surgery procedure

	Baseline N = 48	Three months post-bariatric surgery N = 48	One year post-bariatric surgery N = 48
**General and body composition**			
**Sex** (F/M)		32/12	
**Metabolic syndrome status**, N (%)	36/47 (76.6)^A^	17/41 (41.5)^B^	10/43 (23.3)^C^
**Diabetes status**, N (%)	24 (50.0)^A^	9(18.7)^B^	4(8.3)^C^
**BMI** (kg/m^2^)	49.7 ± 6.50^A^	41.3 ± 5.85^B^	35.1 ± 5.62^C^
**WHR**	0.95 [0.91; 1.02]^A^	0.94 [0.88; 0.99]^A^	0.94 [0.87; 1.01]^A^
**Total fat mass** (in %)	49.4 [43.3; 53.0]^A^	46.0 [38.8; 48.2]^B^	37.0 [28.9; 44.0]^C^
**Visceral fat mass ratio**	19.0 [17.0; 22.0]^A^	15.0 [12.8; 16.0]^B^	12.0 [8.00; 13.0]^C^
**Glycemia, insulin resistance, and antidiabetic medication intake**			
**HbA1c** (%)	5.62 [5.42; 6.66]^A^	5.42 [5.17; 5.77]^B^	5.21 [4.89; 5.64]^C^
**FPG** (mmol/l)	5.50 [5.21; 7.54]^A^	5.25 [4.89; 6.04]^B^	5.00 [4.68; 5.57]^C^
**HOMA-IR**	5.37 [3.16; 8.85]^A^	2.62 [2.12; 2.93]^B^	2.30 [1.07; 3.15]^B^
**Hypertension status**			
**Systolic blood pressure** (mmHg)	132 ± 12.9^A^	120 ± 14.9^B^	120 ± 14.7^B^
**Diastolic blood pressure** (mmHg)	76.6 ± 12.1^A^	68.2 ± 13.0^A^	68.8 ± 11.4^A^
**Dyslipidemia and antihyperlipidemic medication intake**			
**LDL-C** (mmol/l)	3.16 [2.48; 3.81]^A^	2.26 [1.92; 2.83]^B^	2.37 [1.93; 2.91]^B^
**HDL-C** (mmol/l)	1.21 [0.99; 1.40]^A^	1.12 [0.96; 1.35]^A^	1.52 [1.27; 1.59]^B^
**TG** (mmol/l)	1.68 [1.28; 2.15]^A^	1.15 [0.94; 1.46]^B^	0.96 [0.64; 1.30]^C^
**Blood and inflammatory markers**			
**Leukocytes** (Gpt/l)	7.50 [6.70; 8.50]^A^	6.60 [5.50; 8.03]^A^	6.10 [5.20; 7.32]^A^
**LBP** (μg/ml)	20.465[17.89; 24.21]^A^	15.99 [11.75; 17.59]^B^	6.005 [2.36; 14.01]^C^
**sCD14** (ng/ml)	3089 ± 384^A^	3626 ± 323^B^	3243 ± 385^C^
**CRP** (pg/ml)	8.61 [3.87; 18.8]^A^	4.56 [1.49; 10.1]^B^	1.24 [0.43; 3.52]^C^
**Bacterial DNA amount** (in pg per μg extracted whole DNA)	0.64 [0.48; 1.05]^A^	0.48 [0.32; 0.68]^B^	1.24 [0.90; 2.03]^C^

Median and first, as well as third, quartile limits (median [q1; q3]) are shown for non-normally distributed, continuous variables. For normally distributed, continuous variables, data are given in mean ± standard deviation (mean ± SD). For categorical parameters, total numbers (percentage) are shown. Significant p-values are depicted according to group differences: the difference between 2 values in a row with the same letter is non-significant. *Abbreviations*: *BMI* body mass index, *CRP* C-reactive protein, *FPG* fasting plasma glucose, *HbA1c* glycated hemoglobin A1c, *HDL* high-density lipoprotein, *HOMA-IR* homeostatic model assessment for insulin resistance, *LBP* lipopolysaccharide binding protein, *LDL* low-density lipoprotein, *sCD14* soluble CD14, *TG* triglycerides, *WHR* waist to hip ratio

### Reduced bacterial blood load in T2D is ameliorated after bariatric surgery

**Fig. 4:**
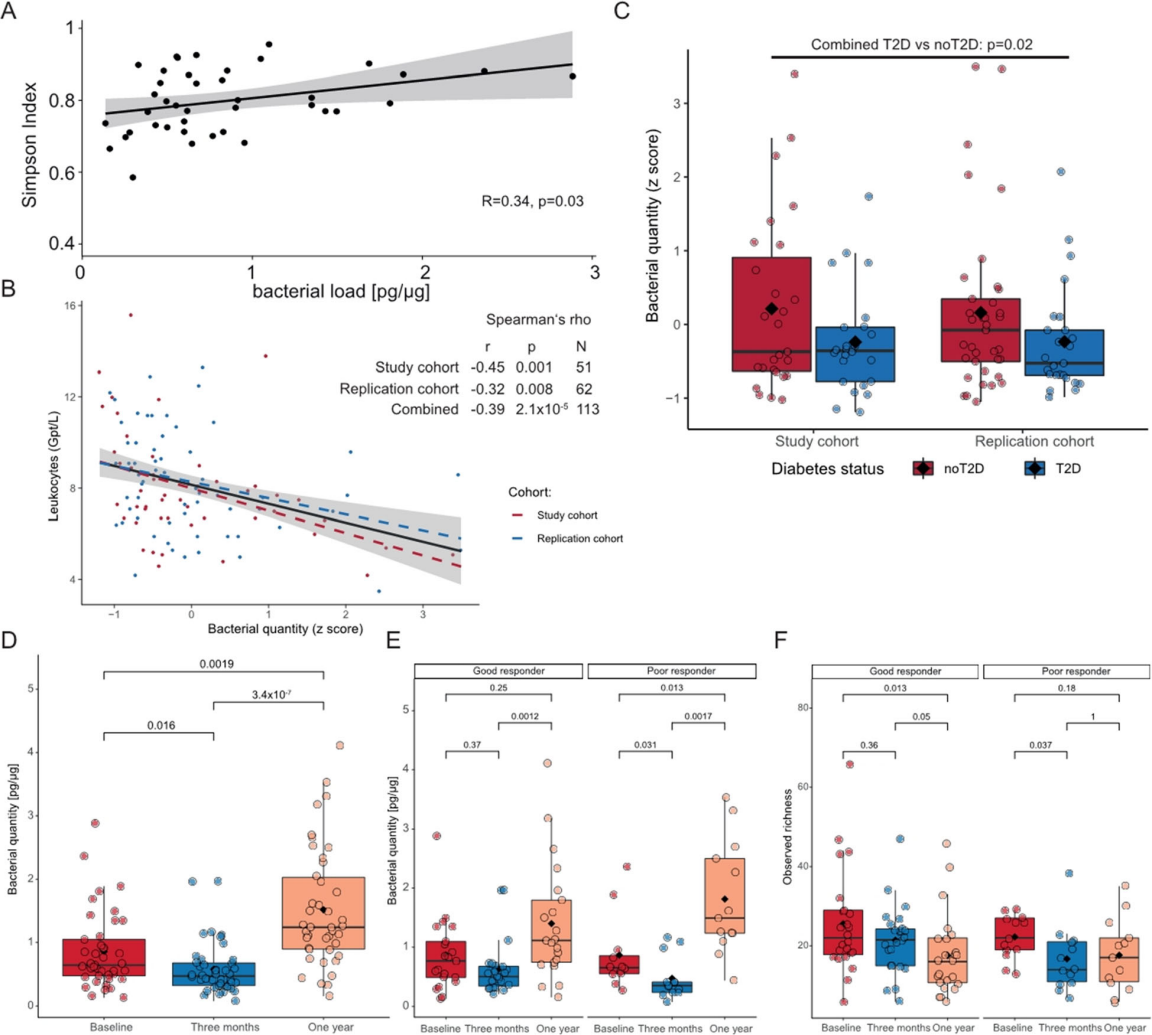
**A** Spearman’s rank correlation of bacterial load at baseline with alpha diversity (Simpson Index), the gray area around the regression line indicates the confidence interval at a confidence level of 95%. **B** Spearman’s rank correlation of bacterial load at baseline with leukocytes in the study and replication cohorts. Colors depict cohorts with red being specific to the study cohort and blue to the replication cohort. Bacterial quantity is standardized within cohorts. The gray area around the regression line indicates the confidence interval at a confidence level of 95%. **C** Blood bacterial in load in T2D vs nonT2D in both study and replication cohorts. Bacterial quantity was standardized prior to statistical analysis. Boxplots are shown with Tukey-whiskers and mean (◆) as well as median. T2D was compared with nonT2D using Wilcoxon signed-rank test after pooling both cohorts. **D** Bacterial quantity in pg per μg extracted DNA over time. The three timepoints are compared using the Kruskal-Wallis test and results are validated via Friedman’s test (Kruskal-Wallis p-value is depicted). Paired samples Wilcoxon signed-rank test is used to compare two groups at once. Only samples with available triplicates in blood bacterial load are used for the comparisons (n = 39). **E** Bacterial quantity dynamics in good and poor responders over time (n = 21 good responders, n = 13 poor responders). The three timepoints are compared using the Kruskal-Wallis test and results are validated via Friedman’s test (Kruskal-Wallis p-value is depicted). Paired samples Wilcoxon signed-rank test is used to compare two groups at once. **F** Diversity dynamics in good and poor responders over time (n = 24 good responders, n = 13 poor responders). The three timepoints are compared using the Kruskal-Wallis test and results are validated via Friedman’s test (Kruskal-Wallis p-value is depicted). Paired samples Wilcoxon signed-rank test is used to compare two groups at once

Bacterial DNA could be measured in 127 blood samples out of 144 samples with available follow-up data at all three timepoints at a quantity ranging from 4.63 to 140.4 fg in 200 μl blood (median = 21.6 fg). Albeit using the same procedure and the same kit, total extracted DNA varied widely between samples spanning from 12.7 to 98.2 ng total DNA/μl blood leading to a corrected range of bacterial DNA quantity within 0.08–4.11 pg per μg total DNA (median = 0.7 pg bacterial DNA per μg total DNA). While bacterial DNA could be detected in negative controls at 0.162 pg per total µg DNA, total DNA in negative controls did not exceed 1.2 ng/μl leading to total bacterial DNA amounts of less than 1.5 fg (median = 0.4 fg). Overall, bacterial blood load was positively associated with bacterial richness at baseline (Fig. 4A) and negatively with leukocytes (Fig. 4B). This observation was further validated in a replication cohort consisting of 62 subjects from the same center (Additional file 1: table S3). Correlation analyses in the replication cohort further underline the negative associations between inflammation and bacterial load and the association between LBP and inflammation as well as obesity (Additional file 1: table S6).

Spanning over all timepoints, bacterial load was negatively correlated with waist circumference, HbA1c, and inflammation markers such as leukocytes as well as markers related to a presumed leaky gut such as LBP (Additional file 1: Fig S6A-D, tables S7, S8). The latter was on the other hand associated with markers of metabolic disease and obesity (Additional file 1: table S7). While these correlations were weak to moderate, the use of Spearman’s rank correlation made them less likely to be influenced by a few high leverage points as shown after removing statistical outliers for bacterial quantity (Additional file 1: Fig. S7A-D). Accordingly, subjects with T2D at baseline displayed lower bacterial quantity in both our study and replication cohorts (Fig. 4C).

Blood bacterial load was decreased 3 months post-bariatric surgery (median bacterial quantity in pg/μg at baseline = 0.032, vs 0.024 at 3 months, p = 0.013, Fig. 4D), but increased significantly at 1 year following surgery (median bacterial quantity in pg/μg at 1 year = 0.062, p-value compared to 3 months = 1.1 × 10^−6^, p-value compared to baseline = 0.002, Fig. 4D) independently of T2D status at baseline (Additional file 1: Fig S8A, B). When checking the changes according to weight loss response, subjects deemed “good responders” and “poor responders” displayed similar blood bacterial load at baseline (median bacterial quantity in pg/μg “good responder” = 0.864 vs “poor responder” = 0.866, p-value = 0.9) but blood bacterial quantity at 1 year increased less dramatically in good responders and was not significant as compared to the increase in blood bacterial quantity in poor responders (fold change 1.6 vs 2.1 in good vs poor responders respectively, Fig. 4E). Bacterial diversity, on the other hand, showed a transient significant decrease at 3 months only to increase again almost to baseline at 1 year in poor responders, while continuously and significantly decreasing in good responders (Fig. 4F).

LBP, expected to reflect host’s response to bacterial DNA, decreased continuously and significantly after bariatric surgery (median LBP in μg/μl at baseline = 20.47 vs 15.99 at 3 months and 6.0 at 1 year after bariatric surgery, p_3MonthvsBaseline_ = 3.3e^−5^, p_1Yearvs3Months_ = 2.46e^−6^, p_1YearvsBaseline_ = 5.8e^−9^) independently of weight loss response or diabetes status at baseline (Additional file 1: Fig. S9A, B). Similarly, leukocytes decreased overtime after bariatric surgery, but less so and non-significantly in subjects with poor response (good responders: p_3MonthvsBaseline_ = 3.3e^−5^, p_1Yearvs3Months_ = 0.14, and p_1YearvsBaseline_ = 6.5e^−4^ respectively; poor responders: p_3MonthvsBaseline_ = 0.67, p_1Yearvs3Months_ = 0.08, and p_1YearvsBaseline_ = 0.16) (Additional file 1: Fig. S9C).

### Metabolic improvement after bariatric surgery is accompanied by early changes in microbial differential abundances in subjects with and without T2D

**Fig. 5:**
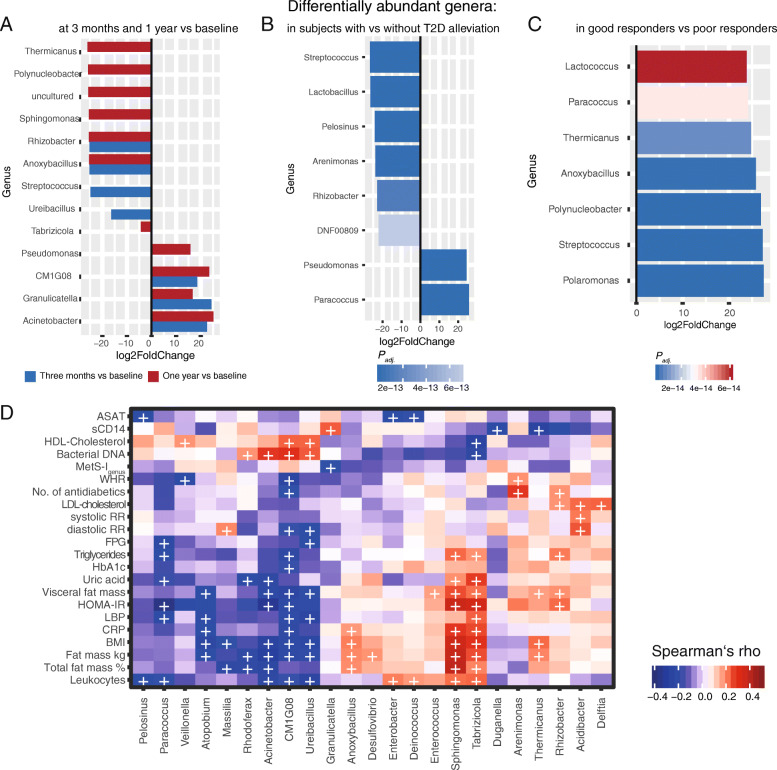
**A** Differentially abundant genera between baseline and 3 months (blue bars) and baseline and 1 year (red) within the complete cohort with available taxonomy at all timepoints (n = 48). Differential abundance is calculated on ASV level abundances using taxonomy after stringent control for contamination at a decontam score 0.5 and reported at the genus level. Only significant differential abundances (*Padj* < 0.01) are reported. **B** Differentially abundant genera in subjects with T2D, who experienced T2D remission as compared to those with persistent T2D (n = 12 in both groups). Color shading represents p-values. **C** Differentially abundant genera in good responders vs poor responders (n = 24 vs 13 respectively). Color shading represents p-values. **D** Spearman’s rank correlations of differentially abundant genera in good responders and subjects with T2D remission with host variables in all timepoints. (+) indicates a significance level < 0.05

Beyond changes in blood bacterial load over time, there were significant and consistent shifts in bacterial genera over the whole cohort between months 3 and 12 after bariatric surgery as compared to baseline. Decreased ASVs belonged to genera such as *Anoxybacillus*, *Rhizobacter*, and *Sphingomonas*, whereas other genera such as *Acinetobacter*, *Granulicatella*, and *Pseudomonas* increased. Comparing shifts at 1 year and baseline showed that most of those seen at 3 months post-bariatric surgery were preserved at 1 year (Fig. 5A).

Moreover, T2D remission following bariatric surgery was associated with significantly lower proportions of *Burkholderiacae*, *Veilonellaceae*, and *Lactobacillaceae* and higher proportions of *Rhodobacteraceae* and *Pseudomonales* at all timepoints combined (Fig. 5B). Genera with observed shifts after bariatric surgery were closely tied to metabolic control: Genera, which had a lower abundance after surgery such as *Anoxybacillus*, *Rhizobacter*, and *Sphingomonas* for example, were significantly positively associated with markers of body composition such as overall and visceral fat mass, BMI, and CRP. Furthermore, significantly positive correlations were observed for aforementioned genera with markers of metabolic control including HOMA-IR, number of antidiabetics, uric acid, triglycerides, and LDL cholesterol (Fig. 5D). As for genera being more abundant after surgery, C*M1G08* and *Acinetobacter* were negatively associated with markers of central obesity and metabolic disease as well as inflammation, while being positively associated with HDL cholesterol and blood bacterial quantity. Contrarily, *Granulicatella* was negatively associated with excess BMI loss, but positively with sCD14. Of note, while *Granulicatella* was higher at 1 year compared to baseline, it seemed to increase at 3 months, while the increase in *GM1G08* and *Acinetobacter* was continuous (Fig. 5A). *Paracoccus—*which was shown to be decreased in T2D at baseline and increased in subjects experiencing T2D remission— was negatively associated with inflammation, FPG, triglycerides, HOMA-IR, and LBP (Fig. 5D).

Good responders displayed an increase in ASVs belonging to *Streptococcaceae* such as *Lactococcus* and *streptococcus* species, *Burkholderiales* such as *Polaromonas* and *Polynucleobacter*, *Rhodobacteraceae* such as *Paracoccus*, and *Bacillaceae* such as *Anoxybacillus* at all timepoints (Fig. 5C). While *Paracoccus* was associated with both decreased inflammation and metabolic impairment, some genera increased in good vs poor response were associated with increased BMI and inflammation such as *Anoxybacillus* or *Thermicanus* overall, but significantly decreased at 1 year when compared to baseline (Fig. 5A). Several genera abundantly observed in good responders and subjects who experienced T2D remission, were initially decreased in T2D, further delineating a putative positive association with good metabolic health (Figs. 3B and 5B–D).

### CARD-FISH allows the visualization of living bacteria in the blood post-bariatric surgery

In order to verify whether increased bacterial quantity post-bariatric surgery is only related to uptake of bacterial DNA or bacteremia, we sought to visualize bacterial cells in a subject, who had undergone bariatric surgery, in whom blood bacterial quantity could be measured at baseline and for whom bacterial sequence data are available. CARD-FISH implementation was unsuccessful in frozen blood samples, but considering blood bacterial quantity increased after surgery over time, we hypothesized that this state is more likely to yield bacterial cell visualization. Because bacteremia and endotoxemia have been associated with chewing and food intake [5, 57, 58], blood samples were also collected 1 h after a mixed meal to increase chances of visualization. Similarly, samples were taken from a healthy athletic control, whose weight has been stable for the last 2 years and who has no known diseases and no medication.

Our CARD-FISH results show the presence of intact bacterial cells in the blood samples from the patient collected before and after mixed meal intake with an abundance of 7.9 × 10^4^ cells ml^−1^ and 1.2 × 10^5^ cell ml^−1^ respectively (Additional file 1: Fig. S10A, B, Additional file 2: Table S8) in support of increased bacteremia after food intake and chewing. In contrast, blood samples from the healthy control revealed no presence of hybridized bacteria, although filter pieces from all filters (3 μm and 0.2 μm) used for cell separation of both the Supernatant and Nycodenz layers were hybridized and imaged (Additional file 1: Fig. S10 C,D). An additional control to certify the CARD-FISH was successful and the lack of hybridized cells in the healthy subject is not due to a technical error, the same control blood sample was deliberately contaminated with *Pseudomonas putida* (DSM6125) (Additional file 1: Fig. S10 E,F). Therein, positively hybridized *P. putida* cells and no other cell morphotypes could be observed, adding evidence that freshly collected blood samples from the healthy control do not contain bacteria at abundances that can be detected by CARD-FISH.

## Discussion

The presence of bacteria and bacterial products in various tissues and their contributions to the local and systemic inflammation have been suggested as novel mechanisms for both development and progression of “non-communicable” metabolic diseases, such as obesity [13–15, 17, 19, 22], T2D [13–15], cardiovascular diseases [17], and cirrhosis [18, 59]. However, studies supporting this hypothesis are mostly limited by the lack of control for contamination and a high overestimation of bacterial DNA quantity, consequently inflating results. In the present work, we therefore quantified and characterized bacterial DNA in the blood of subjects with high metabolic burden undergoing bariatric surgery with an emphasis on including suitable negative controls and subsequently substracting identified contaminant in downstream analyses. The goal was to identify links between metabolism and a putative blood bacterial signature as well as its dynamics over time after bariatric surgery.

After stringent experimental and bioinformatic control for contaminants, we report 2746 features (ASVs) belonging mostly to the phyla *Proteobacteria* and *Firmicutes*, which corroborates previously published results [13, 14, 21, 59, 60]. Furthermore, we evidence quantitative, compositional, and taxonomic signatures associated with markers of metabolic disease and T2D and shifts thereof driven by medication and weight loss intervention. We also provide qualitative and quantitative evidence for the presence of bacterial cells in the blood samples of a subject with obesity, who has undergone bariatric surgery as well as bacteremia after mixed meal intake in comparison to a healthy control.

Our results provide support for the existence of an individually determined core circulatory bacterial signature and microbiome, which reflects disease and intervention with 40% of observed variance in bacterial composition explained by 27 host variables prominently related to inflammation, anthropometric measures, and metabolic health and medication intake. Of the latter, notable associations of bacterial signature were seen for metformin and PPI intake. These results underscore findings from other studies relying on the application of multi-method characterizations including evidence of viability to debunk the notion that the blood is free of viable microorganisms [14, 59–64] as well as studies showing the deeply underrated modulatory effect of medication on host microbial communities and the importance of assessing drug intake in microbial surveys of any niche [65].

Moreover, blood bacteria signature allowed the classification of subjects with and without MetS along their actual clinical group and more accurately so at genus than phylum level. This is in line with recent evidence showing disease-specific tissue signatures in several tissues pertaining to metabolic health as well as cancer [13, 14, 21] and could be related to the particular vulnerability to translocation of specific ingested bacteria in respective diseases. Together, these independent observations reinforce the notion of a predictive circulatory metabolic bacterial signature reminiscent of the described enterotypes [66] of the gut microbiome.

Similarly, we show a specific bacterial signature for T2D characterized by reduced bacterial diversity and an overall reduction in genera belonging to *Firmicutes* and *Proteobacteria*. Diversity scores were negatively correlated with inflammation, visceral adiposity, and uremia, echoing observations of diversity in gut microbial communities in T2D [67]. Although the reported reduction in genera is in line with data from Anhê et al., we did not evidence their reported reduction in *Bacteroidetes* nor the increase in *E. coli* in T2D [13]. While we evidence ASVs belonging to *Bacteroidetes*, their prevalence was very low and was not particularly associated with metabolic control in our cohort. This could be indeed due either to cohort-specific characteristics or our choice in primers. Our data are more in accordance with results from another cohort based in our center. On the other hand, we report several more genera in the blood (314 with 120 core genera), which possibly reflects our choice of whole blood as material compared to plasma samples used by Anhê et al.

A hallmark for T2D in our study was the loss of genera related to *Bacillaceae* and *Bukholderiaceae*. Several of the reduced genera were associated with a healthier metabolic status. Others, such as *Sphingomonas* and *Acidibacter*, were positively associated with BMI and insulin resistance as well as increased blood pressure and antihyperlipidemic medication respectively. Considering subjects with and without T2D at baseline were matched on BMI and did not differ significantly in blood pressure nor lipid levels but were significantly more medicated, these differences could arise from medication effects. This is supported by evidence from Anhê et al. showing increased *Sphingomonas* in subjects with T2D (at least in adipose tissue) and by the fact that these differences disappear after bariatric surgery in our cohort over time, when subjects are taken off their antihyperlipidemic and antihypertensive medication sequentially.

Bariatric surgery, on the other hand, led to swift changes in metabolism and bacterial composition in the blood: Changes occurring after bariatric surgery take place early on and are mostly stable over time, which is in line with available data on the rearrangement of the gut microbiome after bariatric surgery [68]. In addition, subjects who experienced T2D remission showed a significant decrease in several clades associated with cardiometabolic and cardiovascular disease such as *Streptococcaceae* [69], *Veilonellaceae* [70], or *Lactobacillales* [71] which are evidenced to be increased in T2D and under diabetic medication in the gut. In contrast, a few health-related genera increased after bariatric surgery, i.e., genera associated with improved insulin sensitivity and ameliorated lipid status or reduced adiposity at baseline in our cohort. Similarly, subjects who experience T2D remission after bariatric surgery displayed increases in genera seen to be initially decreased in T2D, further delineating a positive putative association of these bacteria with metabolic health.

We further investigated circulating bacterial DNA load: Quantification of 16s rRNA showed that bacterial load was positively associated with bacterial diversity and that the increase of the very small bacterial quantity observed after bariatric surgery reflects a reduced inflammatory tone and an improved metabolic health, which we found intriguing. This was also supported by the observations that (a) bacterial load is reduced in T2D at baseline, also in our independently measured replication cohort; (b) bacterial load was negatively associated with metabolic disease index score at baseline; (c) bacterial load was associated with bacterial composition in the opposite direction dictated by metabolic risk factors such as HbA1c, BMI, and TG; and (d) it increased after bariatric surgery. This is further corroborated by the fact that all associations with bacterial quantity are positive with health markers at each and all timepoints. There was no significant difference in bacterial load at baseline between good and poor responders but poor responders experienced an even more pronounced increase in bacterial load. Unexpectedly, bacterial diversity continuously and significantly decreased in good responders, while in poor responders it significantly dipped around 3 months only to almost recover at 1 year post-bariatric surgery. This was paralleled by similar dynamics for leukocytes in good responders, while poor responders failed to display a significant decrease in leukocytes and inflammation over time. Considering bacterial diversity was still negatively associated with leukocytes at all timepoints, it seems likely that the superior increase in bacterial quantity in poor responders is the driving factor behind the observed increased diversity. The latter, on the other hand and along increased bacterial quantity, could be at least partially responsible for the sustained inflammation seen at 1 year in poor responders. Thus, the increased bacterial quantity after bariatric surgery could be related to increases in gut permeability [72] and transmissibility of oral bacteria after the surgery due to increased pH [73]. Stronger increases thereof in poor responders are possibly related to an even more impaired gut permeability due to sustained obesity [74] as well as increased food intake. This begs the question of the transient reduction in bacterial quantity at 3 months post-bariatric surgery: Considering the origins of bacterial DNA we see are very likely environmental (i.e., food and oral bacteria), bacterial quantity reduction at 3 months could be related to the extreme restriction of food intake, far more pronounced at 3 months than around 1 year after surgery where eating behavior and weight loss are more stable. In support of this, bacterial diversity decreases at 3 months in both good and poor responders only to increase in poor responders again, which could indeed be due to increased food intake. It is important to note that the observations are based on an increase in miniscule amounts of bacterial DNA far from the expected quantity in clinically relevant bacteremia and sepsis.

We would like to acknowledge limitations in our study: Although we control for contamination starting at DNA isolation including filling EDTA vials with PBS via needle butterflies to take production line contamination of medical products into account, we cannot fully exclude contamination via puncture of the skin or other environmental sources. Of note, the highest diligence was used in skin decontamination prior to venipuncture and DNA isolation vials were collected as the last vials (in a sequence of 12 vials). Moreover, we did not observe typical skin bacteria among identified contaminants, making the skin as a source of contamination unlikely. Spurious environmental contamination cannot be fully excluded and might explain the encounter of *Choloroflexi*, *Rhizobacter*, *Limnohabitans*, and *Plactomycetes* as well as *uncultured diatom* in our dataset. While we aimed to reduce these spurious findings as much as possible in a data-driven manner to avoid arbitrary preselection of taxa, even complete subtraction of contaminant AVSs selected via stringent decontam score as done by Poore et al. [21] did not eliminate these taxa from the dataset completely. This commends the development of even better pipelines and bioinformatics tools for decontamination in small bacterial biomass samples. An encouraging observation, nonetheless, is that these taxa do not seem to be particularly relevant for our observed phenotype associations nor does a more stringent decontamination score change the conclusion of our work, further supporting the emergence of disease signatures in circulating bacterial composition.

We evidence circulatory bacterial DNA but can only speculate about its origins: The translocation of bacteria from the gut has been the primary considered mechanism. Although this hypothesis is tempting, we did not validate it by performing gold standard gut permeability tests. Moreover, evidence from the HMP have shown that the blood bacterial signature closely resembles that from the skin and oral cavity [61], further pointing to alternative or additive origins. Considering transmission of oral strains along the gastrointestinal tract is more common than previously recognized [75–77] and that gut rearrangement after bariatric surgery is associated with increased pH in the gut, a shift in bile acid pools [73], and increased PPI prescription especially after RYGB also evident in our cohort [78], we cannot exclude that the change in the circulatory blood bacterial signature is reflective of changes in these, among several other bacterial host niches. The increase in blood bacterial load after bariatric surgery could be seen as underpinning this hypothesis. While our data make the case that other mechanisms override an initial increase in gut permeability, which has been associated with insulin resistance and obesity, to induce weight loss and improve glucose tolerance after bariatric surgery, we are unable to validate this hypothesis at this point.

Finally, the reported correlations on bacterial quantity and those related to bacterial composition with low effect size should be considered with caution. Despite their statistical significance, the biological relevance of these associations might be questionable and commends further independent replications.

Beyond these restrictions and while the evidence for the presence of bacteria in the placenta has been highly controversial, with independent groups refuting these findings, there has been no evidence in the literature that convincingly shows that the blood is a tissue constantly free from bacteria or bacterial genetic material. The limitations of the several studies linking bacteria in the blood with disease have been addressed in the present work further adding to its strengths: Diligent sterile handling of materials and pre-treatment of lab materials with UV light was employed. We further included several negative controls (PBS for extraction and blank controls for PCR), while further accounting for production line contamination of medical products using the same tubes and medical materials used to draw the blood in our subjects. These negative controls were confirmed in agarose gel but were still sequenced alongside the true samples. Moreover, they were actively used to identify possible contaminants in the dataset, which we then excluded using the same Software, that had helped debunk or at least shake the pertinacious notion of a “placenta microbiome” [79, 80]. We further replicated findings on bacterial load in an independent cohort, which was processed and analyzed independently. Moreover, we could substantiate the associations between host metabolic health with individual taxa by several different statistical methods while accounting for the impact of relevant covariates, which has, to our knowledge, never been done previously. Beyond a mere diagnostic tool for metabolic disease, which can be currently achieved more easily and at a lower cost, the current work underpins the notion that the blood is a putative ecological niche. Despite extensive immune control, it remains dynamic and reflects metabolic health, warranting further contaminant conscious rigorous work to delineate mechanistic and exploitable pathways and targets similar to the application of pasteurized bacteria (e.g., *Akkermansia muciniphila*) [81–86] for weight loss and metabolic improvement.

## Conclusions

In summary, after reducing and controlling for both contamination and technical biases, we could detect low amounts of bacterial DNA in the blood of patients, from which we were able to derive a metabolic fingerprint from bacterial DNA composition. We could also observe differences in diversity, amount of bacterial DNA, and bacterial composition between subjects with and without T2D, subjects with or without T2D remission or with varying degrees of weight loss response to bariatric surgery. We further substantiate our findings of bacterial quantity increase after bariatric surgery with imaging of live bacteria. The present work lays a stepping stone and encourages rigorous cross-sectional and longitudinal studies in larger cohorts with both diseased and healthy subjects. These studies should optimally expand over various expertise to provide insights into the functionality and potential role of a “circulatory microbiome” in maintaining health and contributing to the onset and progression of disease.

## Supplementary Information

**Additional file 1.** Contains tables S1-8 and figures S1-10.

**Additional file 2.** Contains tables S2-8.

## Data Availability

The data and code that support the findings of this study are available under “figshare” with the following identifiers: 10.6084/m9.figshare.12885260 10.6084/m9.figshare.12876335 [38, 41]

## Abbreviations

ALAT: Alanin-aminotransferase
ARBs: Angiotensin II receptor blockers
ASAT: Aspartat-aminotransferase
BMI: Body mass index
CRP: C-reactive protein
EBL: Excess BMI loss
eGFR: Estimated glomerular filtration rate
FPG: Fasting plasma glucose
GLP1: Glucagon-like peptide 1
HbA1c: Glycated hemoglobin A1c
HDL: High-density lipoprotein
HOMA-IR: Homeostatic model assessment for insulin resistance
LBP: Lipopolysaccharide binding protein
LDL: Low-density lipoprotein
MetS: Metabolic syndrome
NGT: Normal glucose tolerance
PPI: Proton pump inhibitors
RDA: Redundancy analysis
rRNA: Ribosomal ribonucleic acid
sCD14: Soluble cluster of differentiation 14
T2D: Type 2 diabetes
WHR: Waist to hip ratio
