# Supplementary material for: Circulating bacterial signature is linked to metabolic disease and shifts with metabolic alleviation after bariatric surgery
Source: Genome Med. 2021 Jun 22;13:105. doi: 10.1186/s13073-021-00919-6 (PMC8218394; doi:10.1186/s13073-021-00919-6)
Supplement: Supplementary file 1 — Additional file 1. Contains tables S1-8 and figures S1-10. [file 13073_2021_919_MOESM1_ESM.docx]

## **Additional File 1: Supplementary Materials**

**Circulating bacterial signature is linked to metabolic disease and shifts with metabolic alleviation after bariatric surgery**

Rima M. Chakaroun^1^*#, Lucas Massier^1,2*^, Anna Heintz-Buschart^3,4^, Nedal Said1^1,5^_,_ Joerg Fallmann^8^, Alyce Crane^1^, Tatjana Schütz^1^, Arne Dietrich^6^, Matthias Blüher^1,7^, Michael Stumvoll^1^, Niculina Musat^2^, Peter Kovacs^1,9^

This file contains:

- Supplementary Tables: Pages 1-7
- Supplementary Figures: Pages 8 - 19

## **Supplementary Tables**

| **Table S1 baseline cohort characteristics with initial matching (n=64)** | | | | | |
| --- | --- | --- | --- | --- | --- |
| **Baseline characteristics** | **NGT** | | | **T2D** | **P-value** |
| **N** | 32 | | | 32 |  |
| **General** | | | | | |
| **Sex (F/M)** | 24/8 | | | 24/8 |  |
| **Age (years)** | 40.5 [31.0;45.5] | | | 49 [41.8; 52.2] | **0.025** |
| **BMI (kg/m²)** | 50.3 ± 6.5 | | | 50.2 ± 6.07 | 0.943 |
| **WHR** | 0.91[0.86; 0.95] | | | 0.98 [ 0.92; 1.05] | **0.001** |
| **Active smokers, N (%)** | 4 (12.5%) | | | 8 (25%) | 0.337 |
| **Glycemia, insulin resistance and antidiabetic medication intake** | | | | | |
| **HbA1c (%)** | | 5.38 [5.25;5.51] | | 6.58 [5.95;7.64] | **< 0.001** |
| **FPG (mmol/l)** | | 5.19 [4.86;5.35] | | 7.21 [5.83;9.71] | **< 0.001** |
| **HOMA-IR** | | 3.46 [2.83;5.35] | | 7.30 [4.96;12.5] | **0.001** |
| **Number of anti-T2D drugs (N)** | | 0 | | 2.00 [2.00;3.00] | **< 0.001** |
| **Insulin therapy, N (%)** | | 0 | | 8 (25.0%) | **< 0.001** |
| **Metformin, N (%)** | | 0 | | 23 (71.9%) | **< 0.001** |
| **Sulfonylureas, N (%)** | | 0 | | 2 (6.25%) | **< 0.001** |
| **DPPIV-Inhibitors, N (%)** | | 0 | | 24 (75.0%) | **< 0.001** |
| **GLP1-Agonists, N (%)** | | 0 | | 6 (18.8%) | **< 0.001** |
| **Hypertension status and antihypertensive medication** | | | | | |
| **Hypertension, N (%)** | | | 20 (62.5%) | 26 (81.2%) | 0.164 |
| **Number of anti-HTN drugs, N** | | | 1.00 [0.00;2.00] | 2.00 [0.75;3.25] | 0.287 |
| **Beta-blockers, N (%)** | | | 12 (37.5%) | 12 (37.5%) | 1 |
| **ACE-Inhibitors, N (%)** | | | 7 (21.9%) | 19 (59.4%) | **0.005** |
| **ARBs, N (%)** | | | 7 (21.9%) | 4 (12.5%) | 0.508 |
| **Calcium channel blockers, N (%)** | | | 4 (12.5%) | 7 (21.9%) | 0.508 |
| **Loop diuretics, N (%)** | | | 3 (9.38%) | 7 (21.9%) | 0.302 |
| **K-sparing diuretics, N (%)** | | | 0 | 1 (3.12%) | 1 |
| **Thiazide diuretics, N (%)** | | | 5 (15.6%) | 15 (46.9%) | **0.008** |
| **Systolic blood pressure (mmHg)** | | | 129 ±10.7 | 130 ± 13.3 | 0.753 |
| **Diastolic blood pressure (mmHg)** | | | 75.1 ± 9.99 | 75.7 ±14.2 | 0.852 |
| **Dyslipidemia and antihyperlipidemic medication intake** | | | | | |
| **LDL-C (mmol/l)** | 3.06 ± 0.79 | | | 3.06 ± 0.85 | 0.974 |
| **HDL-C (mmol/l)** | 1.23 [0.99;1.48] | | | 1.15 [0.95;1.28] | 0.170 |
| **TG (mmol/l)** | 1.58 [1.15;1.85] | | | 1.67 [1.42;2.24] | 0.143 |
| **Statin, N (%)** | 1 (3.12%) | | | 8 (25.0%) | **0.014** |
| **Ezetimib, N (%)** | 0 | | | 1 (3.12%) | 1 |
| **Blood and Inflammatory markers** | | | | | |
| **CRP (pg/ml)** | 12.4 [3.47;18.2] | | | 10.6 [6.08;26.1] | 0.294 |

Median and first, as well as third, quartile limits (median [q1; q3]) are shown for non-normally distributed, continuous variables. For normally distributed, continuous variables data are given in mean ± standard deviation (mean ± SD). For categorical parameters, total numbers (percentage) are shown. Significant p-values are depicted in bold. Abbreviations: **ARBs**: Angiotensin II receptor blockers; **BMI**: Body mass index; **CRP**: C-reactive protein; **FPG**: Fasting plasma glucose; **GLP1**: Glukagon-like peptide 1, **HbA1c**: Glycated Hemoglobin A1c; **HDL**: High density lipoprotein; **HOMA-IR:** Homeostatic model assessment for insulin resistance;**LDL**: Low density lipoprotein; **NGT:**Normal glucose tolerance; **T2D:**Type 2 diabetes; **TG**: Triglycerides; **WHR**: Waist to hip ratio.

| **Table S2 Phenotypes of good vs poor responders at baseline and one year post bariatric surgery** | | | | | | | | |  |
| --- | --- | --- | --- | --- | --- | --- | --- | --- | --- |
| **Baseline characteristics** | | **Good Responder** | | | | **Poor Responder** | | **P-value** | |
|  | | **baseline** | | | **One year** | **baseline** | **One year** | **baselines** | **One year** |
| **N** | | 24 | | | | 13 | |  |  |
|  |  | | | | **General** | | | |  |
| **Sex (F/M)** | | 17/7 | | | | 10/3 | |  |  |
| **Age (years)** | | 46.3  ± 8.87 | | | 46.3 ± 8.87 | 48.2  ± 6.14 | 48.2 ± 6.14 | 0.79 |  |
| **BMI (kg/m²)** | | 47.7  ± 5.59 | | | 30.8 2.97 | 51.7  ± 6.58 | 40.9 ± 3.39 | 0.14 |  |
| **WHR** | | 0.96  ± 0.11 | | | 0.91 [0.86;0.98] | 0.93  ± 0.06 | 0.94 [0.90;0.96] | 0.82 |  |
| **Visceral fat ratio** | | 18.8 ± 4.69 | | | 8.00 [6.00;9.25] | 21.2 ± 5.97 | 13.0 [13.0;15.0] | 0.46 |  |
|  |  | | | | **Glycemia, insulin resistance and metabolic syndrome at baseline** | | | |  |
| **T2D Status (Yes)** | | | 11   (45.8%) | | 2 (8.33%) | 9  (69.2%) | 2 (15.4%) | 0.46 | 0.6 |
| **HbA1c (%)** | | | 6.56  ± 1.70 | | 5.13 [4.80;5.44] | 6.24  ± 1.16 | 5.41 [4.94;5.96] | 0.78 | 0.24 |
| **FPG (mmol/l)** | | | 7.28  ± 3.46 | | 5.01 [4.66;5.57] | 7.22  ± 3.07 | 5.33 [4.78;5.81] | 1 | 0.44 |
| **HOMA-IR** | | | 13.8  ± 25.9 | | 1.24 [0.77;2.72] | 7.00  ± 7.55 | 3.20 [2.53;3.53] | 0.63 | **0.02** |
| **Antidiabetis N** | | | 9 (37.5%) | | 2 (8.33%) | 9 (69.2%) | 2 (15.4%) | 0.27 | 0.60 |
| **MetS Status** | | | 19 (79.2%) | | 4 (18.2%) | 9 (69.2%) | 4 (33.3%) | 1 | 0.60 |
|  |  | | | | **Hypertension status and antihypertensive medication** | | | |  |
| **Systolic RR (mmHg)** | | | | 129  ± 13.5 | 118 ± 15.9 | 137 ± 12.5 | 124 ±13.2 | 0.30 | 0.23 |
| **Diastolic RR (mmHg)** | | | | 76.0  ± 12.3 | 67.3 ± 10.7 | 76.0  ± 11.8 | 69.9 ± 11.7 | 1 | 0.5 |
|  |  | | | | **Dyslipidemia and antihyperlipidemic medication intake** | | | |  |
| **LDL-C (mmol/l)** | | 3.18  ± 1.04 | | | 2.08 [1.83;2.68] | 3.24  ± 0.69 | 2.67 [2.42;2.97] | 0.98 | **0.03** |
| **HDL-C (mmol/l)** | | 1.26  ± 0.40 | | | 1.54 [1.33;1.74] | 1.25  ± 0.25 | 1.52 [1.35;1.55] | 0.99 | 0.44 |
| **TG (mmol/l)** | | 1.84  ± 0.84 | | | 0.86 [0.62;1.08] | 1.75  ± 0.59 | 1.21 [1.01;1.50] | 0.93 | **0.03** |
| **Statin, N (%)** | | 4  (16.0%) | | | 4 (16.7%) | 3  (23.1%) | 3 (23.1% | 0.96 | 0.67 |
|  |  | | | | **Blood and Inflammatory markers** | | | |  |
| **Haemoglobin (g/dl)** | | 13.9  ± 1.18 | | | 13.1 ±1.21 | 13.7  ± 0.64 | 13.0 ± 1.21 | 0.88 | 0.83 |
| **Leucocytes (Gpt/l)** | | 7.99  ± 2.45 | | | 6.23 ±1.58 | 7.65  ± 1.75 | 6.84 ± 1.89 | 0.88 | 0.33 |
| **CRP (pg/ml)**  **Bacterial load fg/ng** | | 11.7 ± 9.86  0.86 ± 0.62 | | | 0.94 [0.38;1.60]  1.11 [0.74;1.80] | 14 ± 11.8  0.87 ± 0.62 | 3.41 [1.12;5.36]  1.49 [1.24;2.50] | 0.95  1 | **0.018**  0.14 |

Continuous variables data are given in mean ± standard deviation. Median and first, as well as third, quartile limits (median [q1; q3]) are shown for non-normally distributed. For categorical parameters, total numbers (percentage) are shown. Significant p-values are depicted in bold. Abbreviations:  **BMI**: Body mass index; **CRP**: C-reactive protein; **FPG**: Fasting plasma glucose;  **HbA1c**: Glycated Hemoglobin A1c; **HDL**: High density lipoprotein; **HOMA-IR:** Homeostatic model assessment for insulin resistance;**LDL**: Low density lipoprotein; **NGT:**Normal glucose tolerance; RR: Blood pressure (Riva Rocci)  **T2D:**Type 2 diabetes; **TG**: Triglycerides; **WHR**: Waist to hip ratio.

| **Table S3 Replication Cohort characteristics** | | | | |
| --- | --- | --- | --- | --- |
| **Baseline characteristics** | **NGT** | | **T2D** | **P-value** |
| **N** | 37 | | 25 |  |
| **General** | | | | |
| **Sex (F/M)** | 27/10 | | 16/9 | 0.57 |
| **Age (years)** | 44 ± 11 | | 53 ± 11 | **0.003** |
| **BMI (kg/m²)** | 46.28 ± 5.63 | | 48.46 ± 7.4 | 0.38 |
| **Glycemia, insulin resistance and lipid metabolism** | | | | |
| **FPG (mmol/l)** | | 5.41 ± 1.07 | 7.06 ± 1.93 | **0.001** |
| **HOMA-IR** | | 4.70 ± 3.91 | 9.23 ± 12.68 | 0.11 |
| **Triglycerides (mmol/l)** | | 1.28 ± 0.76 | 2.25 ± 2.22 | 0.05 |
| **HDL-Cholesterol (mmol/l)** | | 1.16 ± 0.34 | 0.98 ± 0.28 | **0.04** |
| **LDL-Cholesterol (mmol/l)** | | 2.84 ± 0.72 | 2.57 ± 0.86 | 0.2 |
| **Inflammatory markers** | | | | |
| **Leukocytes (Gpt)** | | 8.08 ± 2.24 | 8.54 ± 2.33 | 0.44 |
| **LBP** | | 11.72 ± 4.00 | 14.33 ± 6.26 | 0.07 |

Data are given in mean ± standard deviation (mean ± SD). For categorical parameters, total numbers (percentage) are shown. Significant p-values are depicted in bold. **Abbreviations**: **BMI**: Body mass index; **CRP**: C-reactive protein; **FPG**: Fasting plasma glucose; **HbA1c**: Glycated Hemoglobin A1c; **HOMA-IR:** Homeostatic model assessment for insulin resistance;**LBP**: Lipopolysaccharide binding protein

**Table S4: Envfit output for 27 variables on genera level RDA**

|  | RDA1 | RDA2 | R² | Adj. P(r) |
| --- | --- | --- | --- | --- |
| Variables | | | | |
| BMI | 1.00000 | 0.00007 | 0.2219 | 0.001*** |
| Systolic blood pressure | 0.73835 | -0.67441 | 0.0158 | 0.487 |
| eGFR | 0.09266 | -0.99570 | 0.0053 | 0.780 |
| CRP_us | 0.81536 | -0.57896 | 0.0667 | 0.025* |
| HbA1c | 0.50241 | -0.86463 | 0.0753 | 0.027* |
| HDL- cholesterol | -0.99377 | 0.11144 | 0.0963 | 0.016* |
| LDL-cholesterol | -0.91281 | 0.40839 | 0.0231 | 0.306 |
| triglycerides | 0.88166 | 0.47189 | 0.1560 | 0.001*** |
| Total fat mass in % | 0.96126 | -0.27565 | 0.2090 | 0.001*** |
| Visceral fat rating | 0.79766 | -0.60310 | 0.0719 | 0.030* |
| Nr of antidiabetics | 0.99683 | 0.07956 | 0.0541 | 0.069 **^.^** |
| Nr of antihypertensives | -0.65734 | -0.75359 | 0.0008 | 0.964 |
| Nr of antihyperlipidemics | -0.07182 | 0.99742 | 0.0135 | 0.514 |
| White blood cells count | 0.87696 | -0.48055 | 0.2531 | 0.001*** |
| Age | -0.75498 | -0.65575 | 0.0653 | 0.034* |
| Bacterial quantity | -0.99870 | 0.05102 | 0.1788 | 0.001*** |
| Waist-to-hip-ratio | -0.24996 | -0.96826 | 0.0860 | 0.015* |
|  | | | | |
| Diabetes Status _ No | -4.5303 | 5.4529 | 0.0630 | 0.002** |
| Diabetes Status _ Yes | 16.2677 | -19.5809 |  |  |
| Sex_female | 4.2702 | 3.6557 | 0.0318 | 0.039* |
| Sex_male | -12.3180 | -10.5452 |  |  |
| Metformin intake_No | -4.0974 | -0.8525 | 0.0380 | 0.022* |
| Metformin intake_Yes | 25.4624 | 5.2977 |  |  |
| PPI intake No | -16.5911 | 7.6883 | 0.0674 | 0.001*** |
| PPI intake Yes | 9.5917 | -4.4448 |  |  |
| Timepoint_baseline | 20.2299 | 9.4054 | 0.2184 | 0.001*** |
| Timepoint_one_year | -31.7231 | -7.1606 |  |  |
| Timepoint_three_months | 16.9608 | 0.2972 |  |  |
| Metabolic syndrome No | -10.5658 | 7.8723 | 0.0886 | 0.001*** |
| Metabolic Syndrome Yes | 15.4622 | -11.5205 |  |  |
| T2D Alleviation_ Yes | 14.2149 | -2.4049 | 0.0278 | 0.068 |
| T2D Alleviation_No | -5.4523 | 0.9224 |  |  |
| EBL_ good responder | -4.0761 | 7.9539 | 0.0389 | 0.082 |
| EBL_ medium_responder | 9.7610 | -2.1739 |  |  |
| EBL_ poor_responder | 1.8058 | -13.0851 |  |  |
| Surgical procedure_RYGB | -0.6923 | 21.583 | 0.0147 | 0.220 |
| Surgical procedure_Sleeve | 56.641 | -176.590 |  |  |

**Table S5: Envfit output for 27 variables on ASV level RDA**

|  | RDA1 | RDA2 | R² | Adj. P(r) |
| --- | --- | --- | --- | --- |
| Variables | | | | |
| BMI | 0.99700 | -0.07735 | 0.2549 | 0.001*** |
| Systolic blood pressure | 0.39658 | 0.91800 | 0.0862 | 0.016* |
| eGFR | 0.14059 | 0.99007 | 0.0328 | 0.181 |
| CRP_us | 0.92738 | -0.37411 | 0.0580 | 0.059 |
| HbA1c | 0.54979 | 0.83530 | 0.0828 | 0.026* |
| HDL- cholesterol | -0.92841 | 0.37155 | 0.1038 | 0.010** |
| LDL-cholesterol | -0.72935 | -0.68414 | 0.0276 | 0.255 |
| triglycerides | 0.87812 | -0.47844 | 0.1622 | 0.002** |
| Total fat mass in % | 0.99994 | 0.01054 | 0.1999 | 0.001*** |
| Visceral fat rating | 0.98608 | -0.16627 | 0.0635 | 0.043* |
| Nr of antidiabetics | 0.93161 | 0.36347 | 0.0737 | 0.030* |
| Nr of antihypertensives | 0.03295 | -0.99946 | 0.0124 | 0.557 |
| Nr of antihyperlipidemics | -0.17617 | -0.98436 | 0.0023 | 0.906 |
| White blood cells count | 0.84021 | 0.54226 | 0.3037 | 0.001*** |
| Age | -0.97574 | -0.21895 | 0.0343 | 0.171 |
| Bacterial quantity | -0.97013 | -0.24259 | 0.2082 | 0.001*** |
| Waist-to-hip-ratio | 0.99994 | 0.01054 | 0.1999 | 0.001*** |
|  | | | | |
| Diabetes Status _ No | -4.7134 | -6.2159 | 0.0829 | 0.001*** |
| Diabetes Status _ Yes | 16.9256 | 22.3208 |  |  |
| Sex_female | 3.7401 | 0.2824 | 0.0154 | 0.214 |
| Sex_male | -10.7886 | -0.8147 |  |  |
| Metformin intake_No | -4.2775 | 0.4924 | 0.0437 | 0.013* |
| Metformin intake_Yes | 26.5815 | -3.0602 |  |  |
| PPI intake No | -14.8085 | 6.8333 | 0.0583 | 0.002*** |
| PPI intake Yes | 8.5612 | -3.9505 |  |  |
| Timepoint_baseline | 21.4085 | -6.1564 | 0.2333 | 0.001*** |
| Timepoint_one_year | -31.0180 | 9.5650 |  |  |
| Timepoint_three_months | 15.3955 | -5.0725 |  |  |
| Metabolic syndrome No | -9.7827 | -5.1334 | 0.0678 | 0.001*** |
| Metabolic Syndrome Yes | 14.3161 | 7.5123 |  |  |
| T2D Alleviation_ Yes | 12.3703 | -2.7700 | 0.0234 | 0.108 |
| T2D Alleviation_No | -4.7448 | 1.0625 |  |  |
| EBL_ good responder | -3.8266 | -6.2655 | 0.0468 | 0.052 |
| EBL_ medium_responder | 10.0702 | -6.9565 |  |  |
| EBL_ poor_responder | 1.1815 | 15.2199 |  |  |
| Surgical procedure_RYGB | -0.8286 | -0.2355 | 0.0023 | 0.804 |
| Surgical procedure_Sleeve | 6.7793 | 1.9271 |  |  |

| **Table S6 Spearman’s rank correlations between bacterial load and LBP with host variables in the replication cohort** | | | |
| --- | --- | --- | --- |
| **Covariate 1** | **Covariate 2** | **Beta-coefficient** | **P-value** |
| Bacterial load in pg/µg isolated DNA | **Circ. TNF-α** | **-0.26** | **0.04** |
|  | LBP | -0.138 | 0.29 |
|  | HbA1c (%) | -0.12 | 0.33 |
|  | HOMA-IR | -0.20 | 0.11 |
|  | **Leukocytes count (in Gpt/l)** | **-0.38** | **<0.001** |
| LBP | **BMI** | **0.32** | **0.008** |
|  | **CRP** | **0.35** | **0.005** |
|  | **HOMA-IR** | **0.28** | **0.03** |
|  | HbA1c | 0.24 | 0.06 |
|  | **Leukocytes count** | **0.29** | **0.02** |

**Abbreviations**: **BMI**: Body mass index; **CRP**: C-reactive protein; **FPG**: Fasting plasma glucose; **HbA1c**: Glycated Hemoglobin A1c; **HOMA-IR:** Homeostatic model assessment for insulin resistance;**LBP**: Lipopolysaccharide binding protein, **TNF- α**: Tumor necrosis factor alpha

| **Table S7 Spearman’s rank correlations between bacterial load and LBP with host variables in the study cohort** | | | | |
| --- | --- | --- | --- | --- |
| **Timepoint** | **Covariate 1** | **Covariate 2** | **Beta-coefficient** | **P-value** |
| **baseline** | Bacterial load in fg/ng isolated DNA | Observed microbial richness | 0.33 | <0.05 |
|  |  | Leukocytes | -0.45 | <0.01 |
| **All tim points** |  | HbA1c (%) | -0.24 | <0.01 |
|  |  | Uric acid in mmol/l | -0.24 | <0.01 |
|  |  | Total fat mass (kg) | -0.19 | <0.05 |
|  |  | Total weight (kg) | -0.21 | <0.05 |
|  |  | Waist circumference | -0.21 | <0.05 |
|  |  | Platelet count | -0.31 | <0.001 |
|  |  | Leukocytes count (in Gpt/l) | -0.38 | <0.001 |
|  |  | LBP | -0.274 | <0.05 |
|  | LBP | BMI | 0.47 | < 0.001 |
|  |  | WHR | 0.34 | <0.05 |
|  |  | HOMA-IR | 0.32 | <0.05 |
|  |  | HbA1c | 0.4 | <0.05 |
|  |  | Number of antidiabetics | 0.35 | <0.05 |
|  |  | Leukocytes count | 0.36 | <0.05 |

**Table S8 Spearman’s rank correlations between bacterial load and host variables in the study cohort at baseline and the replication cohort (for which only baseline is available)**

|  | Study cohort | |  | Replication cohort | |  | Combined | | |  | |
| --- | --- | --- | --- | --- | --- | --- | --- | --- | --- | --- | --- |
|  | ρ | p | N | ρ | p | N | ρ | p | N | |  |
| **BMI** | -0.074 | 0.612 | 50 | -0.087 | 0.502 | 62 | -0.089 | 0.351 | 112 | |  |
| **HOMA-IR** | -0.027 | 0.872 | 37 | -0.216 | 0.106 | 57 | -0.151 | 0.146 | 94 | |  |
| **Triglycerides** | 0.346* | 0.013 | 51 | -0.325* | 0.013 | 58 | -0.019 | 0.842 | 109 | |  |
| **LBP** | -0.122 | 0.446 | 41 | -0.167 | 0.224 | 55 | -0.138 | 0.181 | 96 | |  |
| **Leukocytes** | **0.450**** | **0.001** | **51** | **-0.334**** | **0.008** | **62** | **-0.388**** | **2.1x10-5** | **113** | |  |
| **Fasting plasma glucose (mmol/l)** | 0.112 | 0.486 | 41 | -0.173 | 0.178 | 62 | -0.048 | 0.632 | 103 | |  |
| **Cholesterol (mmol/l)** | 0.194 | 0.174 | 51 | 0.016 | 0.908 | 58 | 0.091 | 0.348 | 109 | |  |
| **HDL-cholesterol** | -0.319* | 0.022 | 51 | -0.028 | 0.834 | 58 | -0.158 | 0.101 | 109 | |  |
| **LDL-cholesterol** | 0.178 | 0.212 | 51 | 0.148 | 0.266 | 58 | 0.158 | 0.101 | 109 | |  |


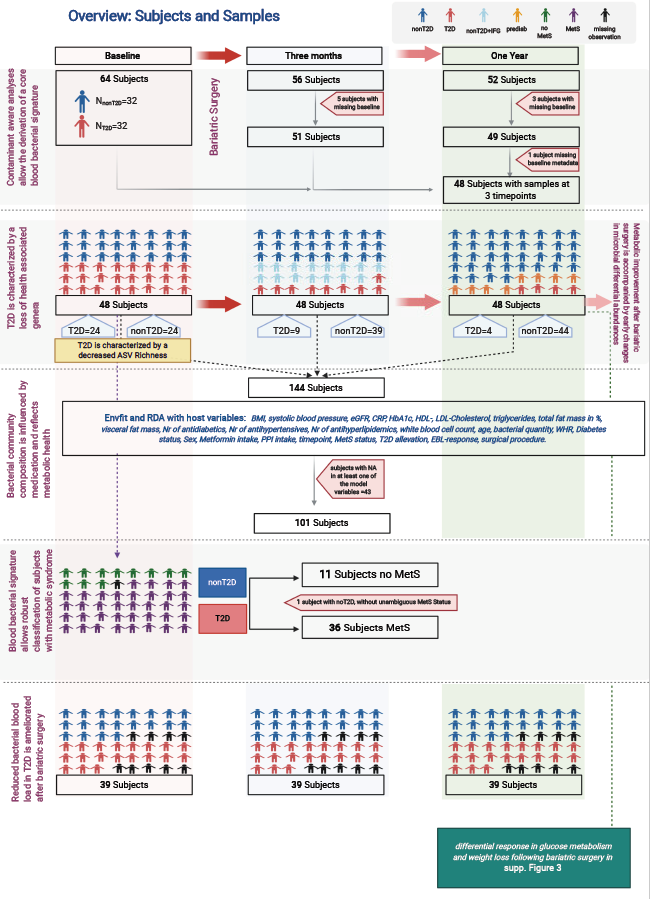


**Figure S1** Flowchart with number of subjects / Datasets and ASVs in each reported analyses

**Figure S1**

**Figure S2**


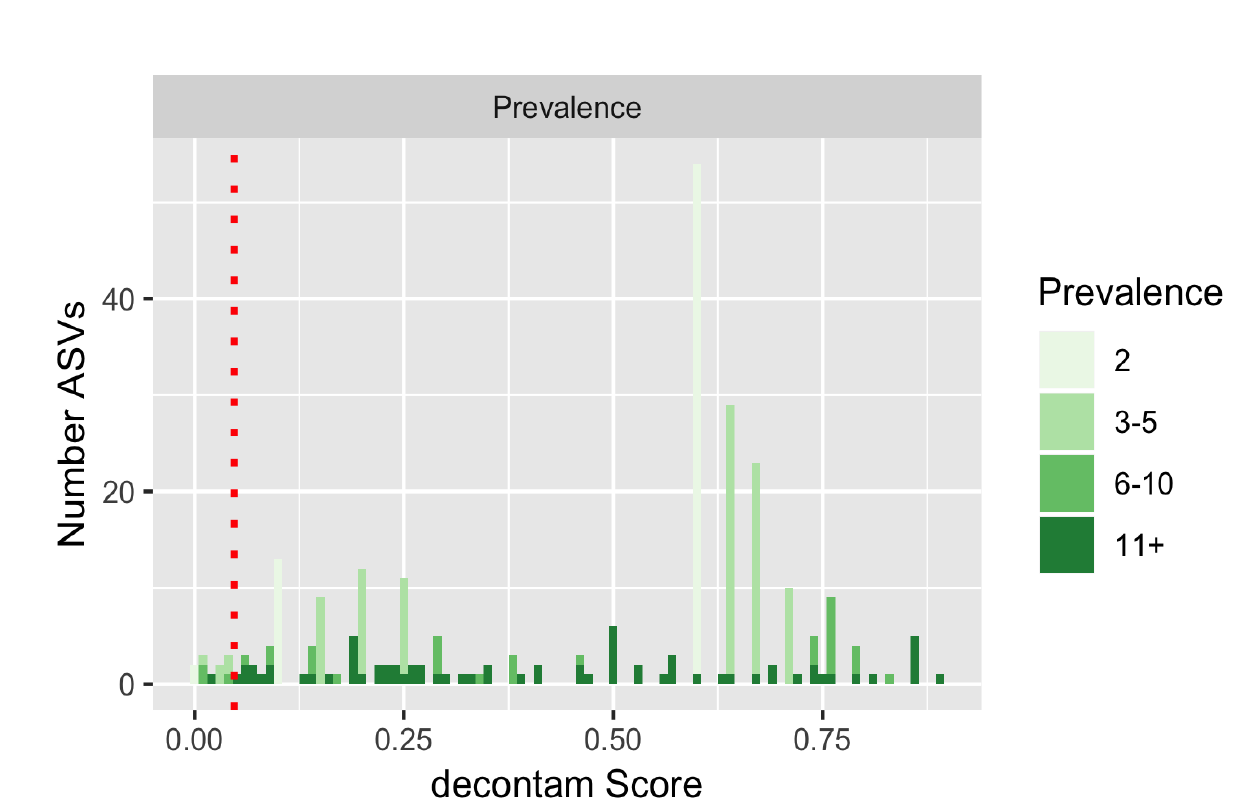


##### **Figure S2** Number of ASVs with respective prevalence along Decontam scores: The contaminant scores show a bimodal distribution with low scores for lower prevalence ASVs around 0.1, higher prevalence ASVs (in which we are confident) start to rise beyond the threshold of 0.175.

**Figure S3**


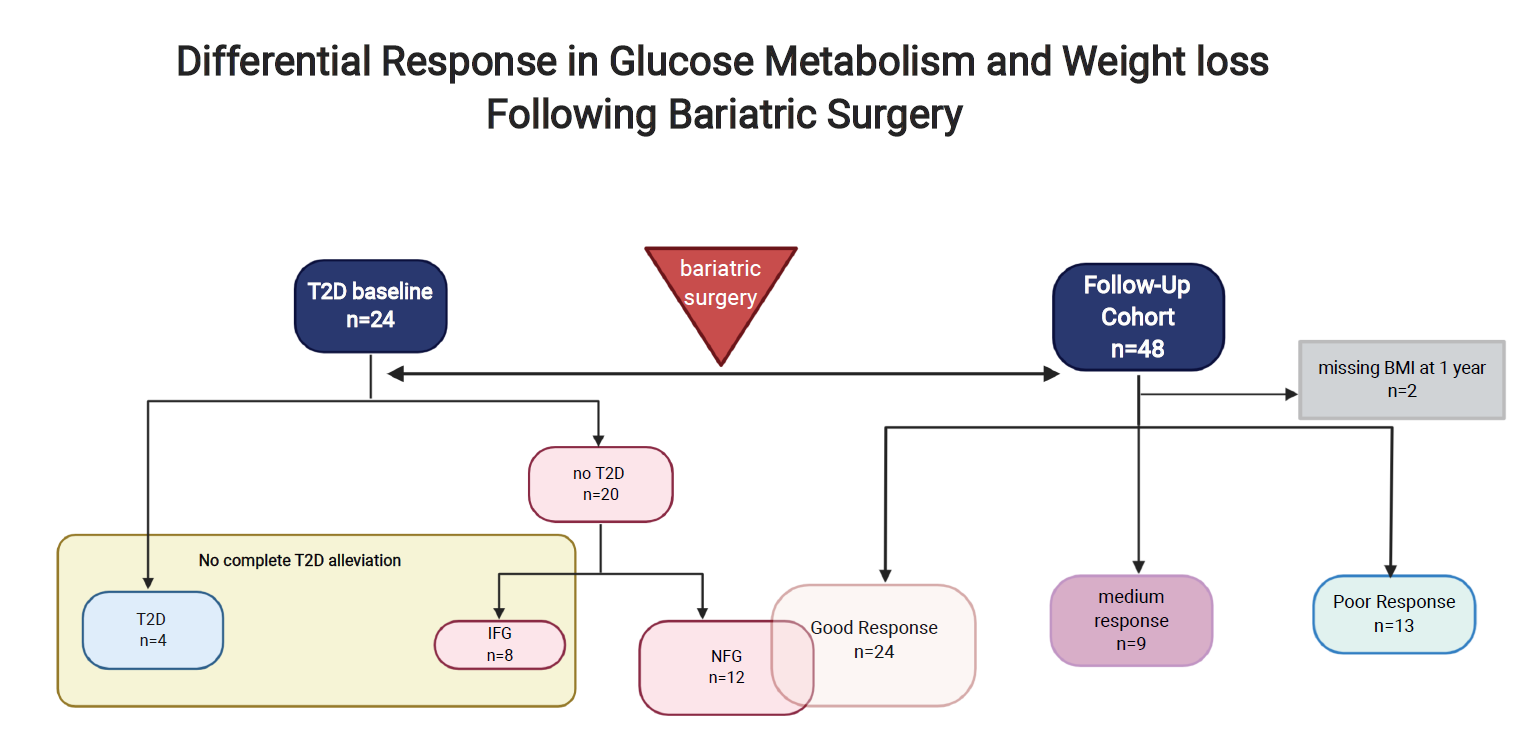


**Figure S3** flowchart showing T2D alleviation and response after bariatric surgery

**Figure S4**


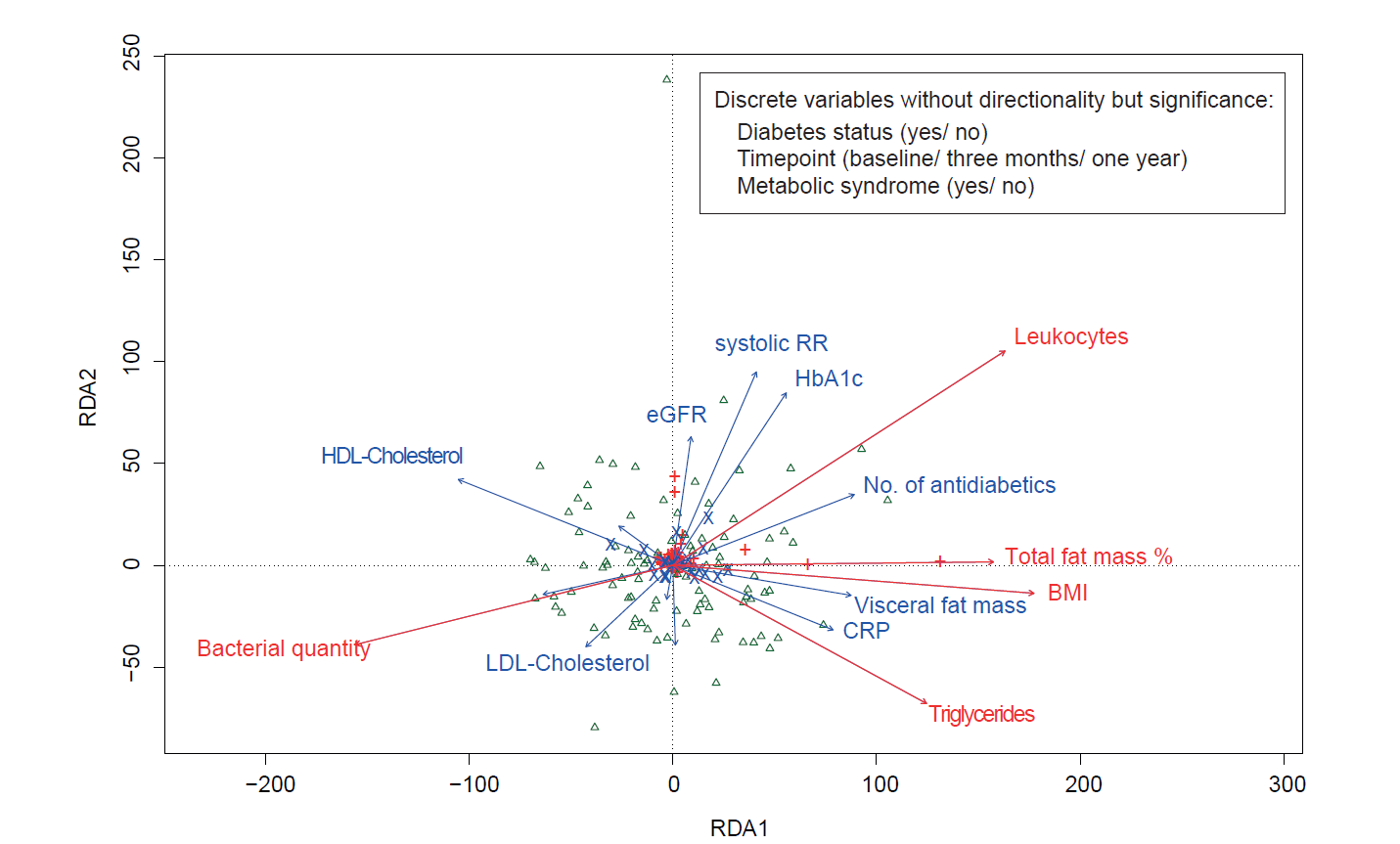


**Figure S4** correlation Triplot fitted site scores as linear combinations of the environmental variables and constraints (explanatory variables), highly significant variables with p< 0.002 are shown in red. First two canonical axes are visualized. Species scores are shown as red crosses, samples are shown as green triangles; Arrowheads depict significant explanatory variables and their directional contribution to the variance in the dataset.

**Figure S5**


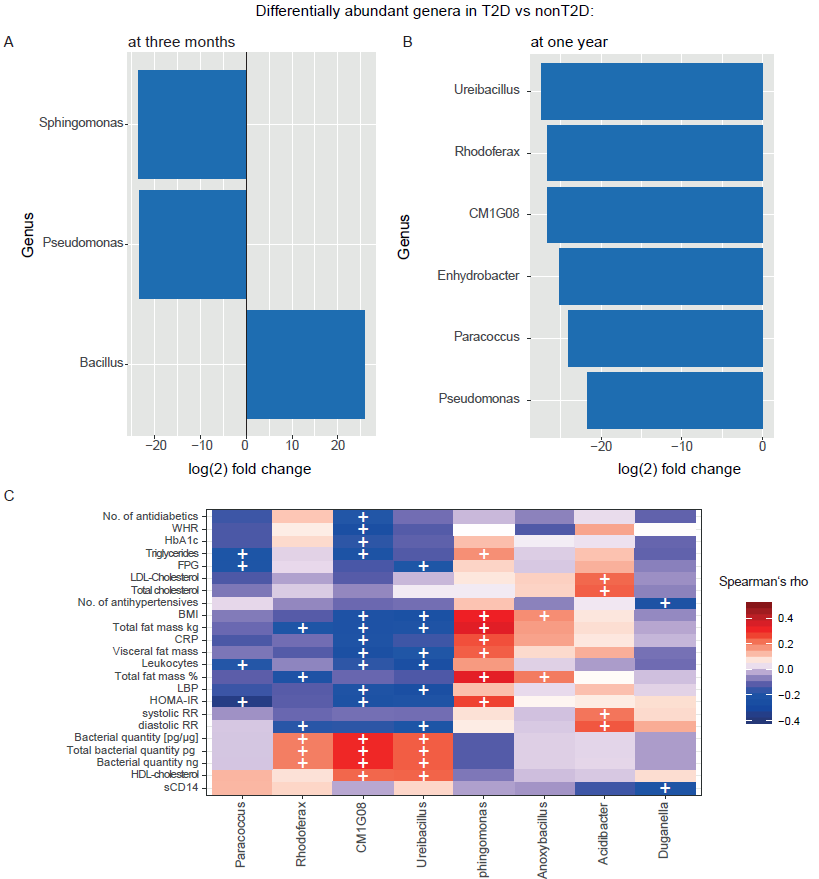


**Figure S5 (A)** Differentially abundant genera in subjects with T2D (n=9) compared to subjects without T2D (n=39) at 3 months post bariatric surgery. Differential abundance is calculated at ASV level using taxonomy after stringent control for contamination at a decontam score of 0.5 and reported at genus level.  **(B)** Differentially abundant genera in subjects with T2D (n=4) compared to subjects without T2D (n=44) at one year post bariatric surgery. Differential abundance is calculated on ASV level using taxonomy after stringent control for contamination at a decontam score of 0.5 and reported at genus level.  Color shading represents p-values. **(C)** Spearman’s rank correlations of relative selected genera abundance with host parameters at 3 months and one year post bariatric surgery. Selection included genera seen to be differentially abundant between groups (i.e. T2D, good/poor responders and T2D alleviation vs no T2D alleviation). Only genera are shown with at least one significant correlation with host markers. (+) refers to p-value < 0.05. Color represents correlation strength (Rho) according to color legend.

**Figure S6**


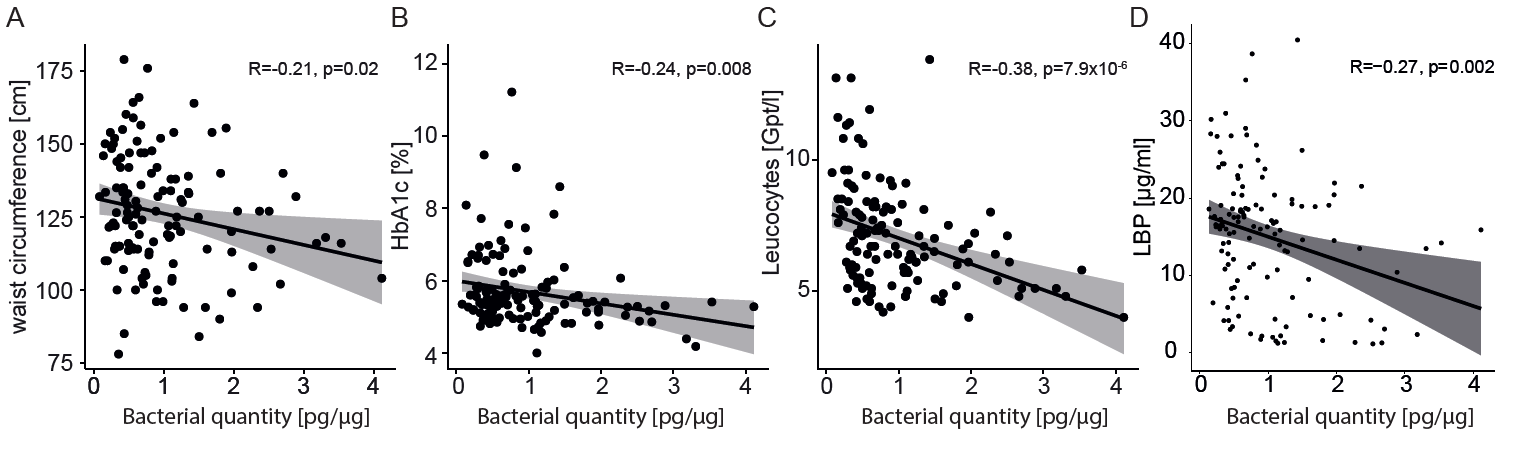


**Figure S6 (A-D)** Spearman’s rank correlations of bacterial quantity in pg per total µg extracted DNA with markers or inflammation and metabolic disease over all timepoints.The grey area around regression line indicates the confidence interval at a confidence level of 95%.

**Figure S7**


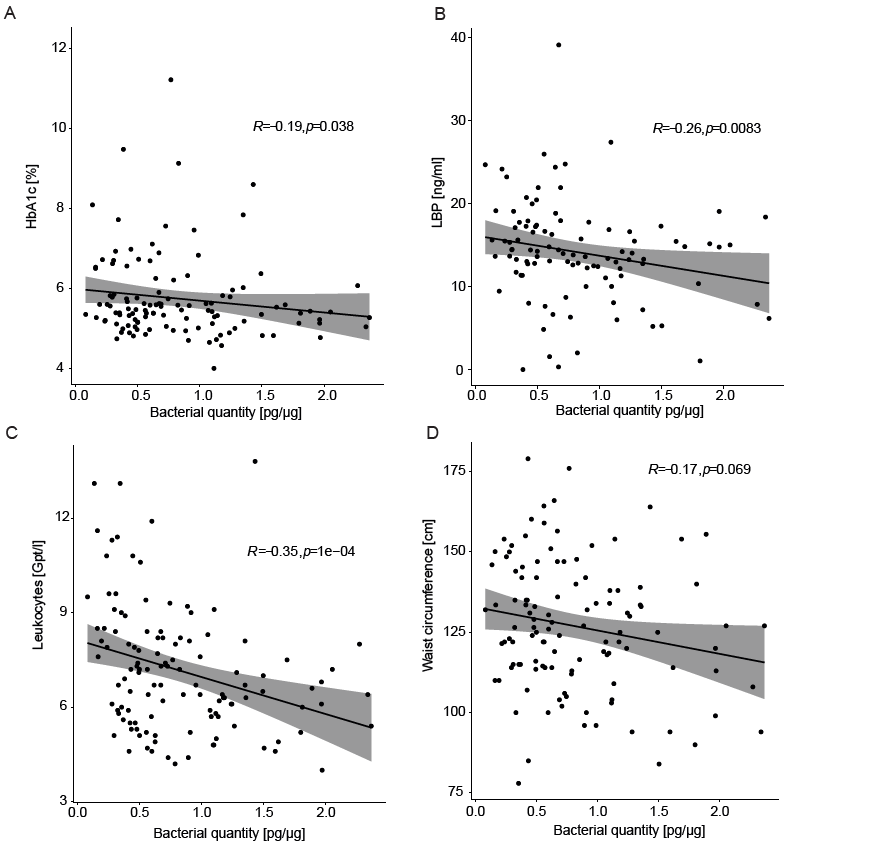


**Figure S7 (A-D)** Spearman’s rank correlations of bacterial quantity in pg per total µg extracted DNA with markers or inflammation and metabolic disease over all timepoints after removing statistical outliers for bacterial quantity. The grey area around regression line indicates the confidence interval at a confidence level of 95%.

##### **Figure S8**


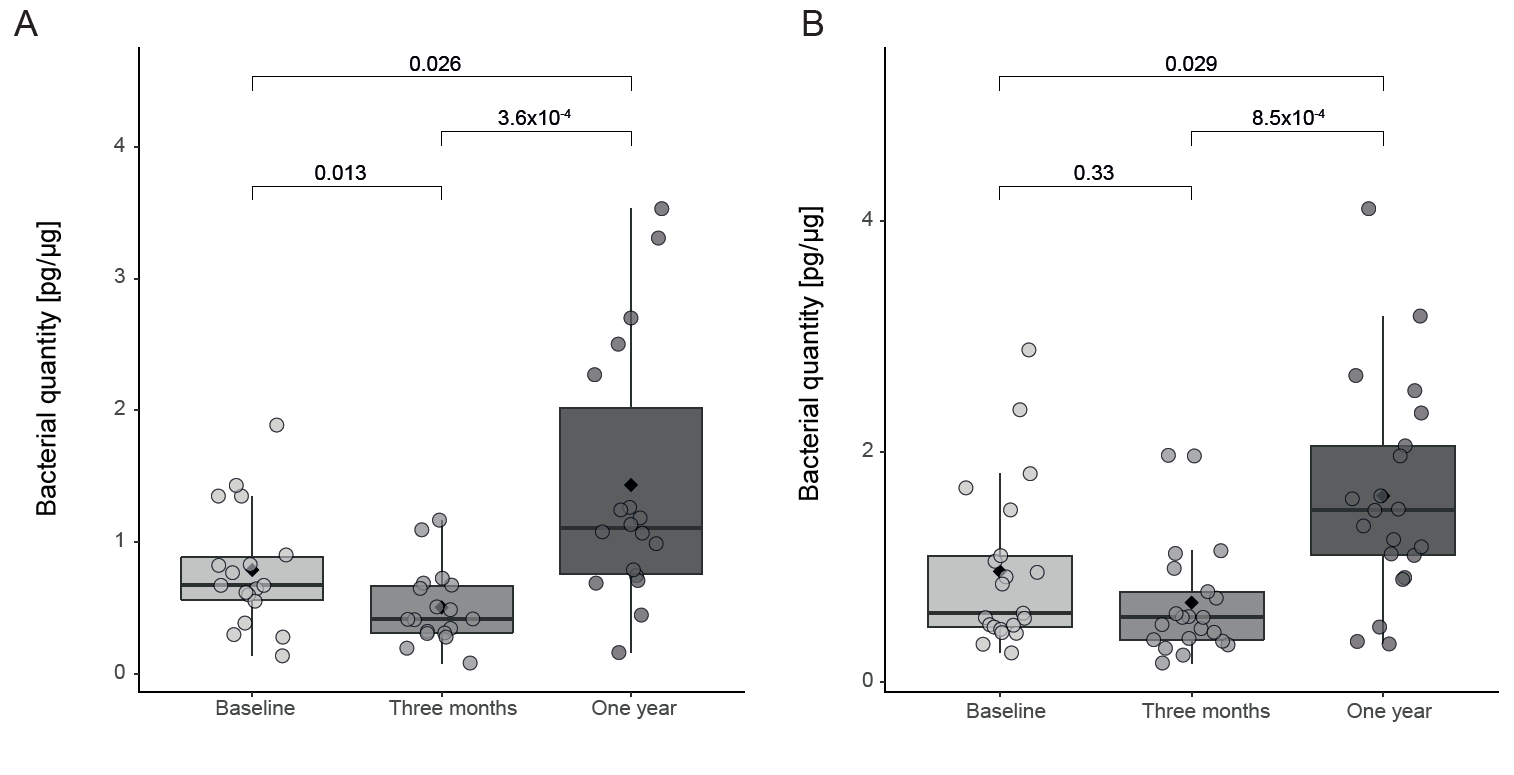


##### **Figure S8** bacterial quantity over time according to T2D and non T2D **(A,B)** respectively. Four samples with missing baseline bacterial quantity were eliminated. Boxplots are shown with Tukey-whiskers and mean (◆) as well as median. The three timepoints are compared using Kruskal-Wallis test and results are validated via Friedman’s test (Kruskal-Wallis p-value is depicted). Paired samples Wilcoxon signed-rank test is used to compare two groups at once.

##### **Figure S9**


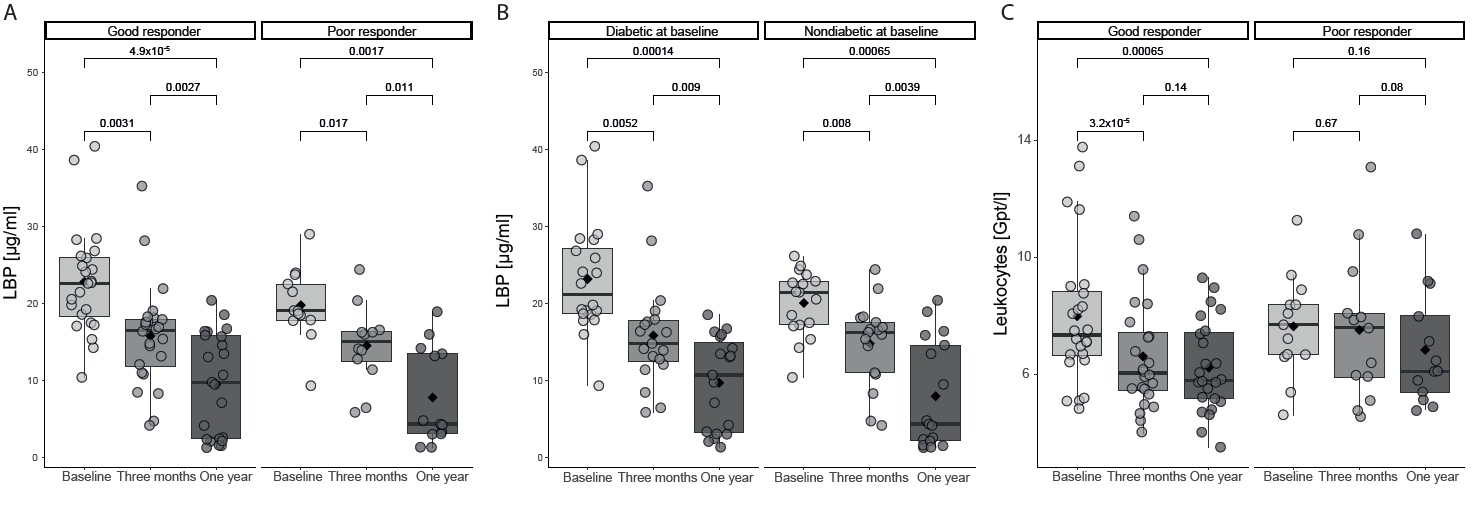


##### **Figure S9 (A)** Changes in LBP over time in good vs poor responders. **(B)** Changes in LBP over time on subjects with and without T2D at baseline **(C)** Changes in leukocytes over time in good vs poor responders. Boxplots are shown with Tukey-whiskers and mean (◆) as well as median. The three timepoints are compared using Kruskal-Wallis test and results are validated via Friedman’s test (Kruskal-Wallis p-value is depicted). Paired samples Wilcoxon signed-rank test is used to compare two groups at once.

**Figure S10**


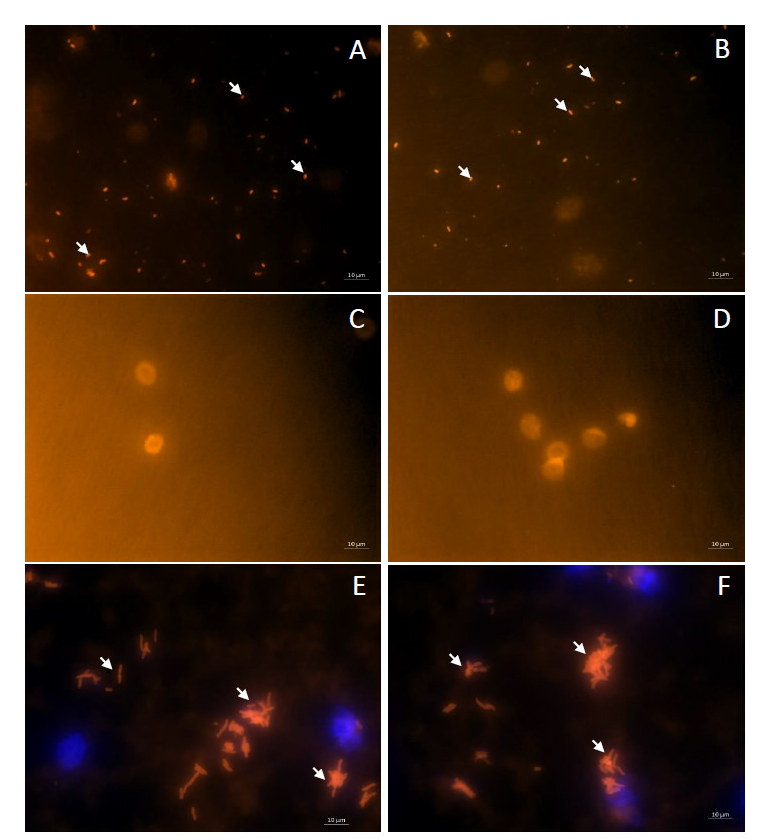


**Figure S10** Representative epifluorescence micrographs obtained by green light excitation and UV excitation of hybridized blood samples using CARD-FISH with HRP-labelled EUB338 probe (in red) and DAPI staining (in blue). (**A-B**) Positively hybridized bacterial cells (arrows) in fresh blood collected before mixed meal intake (**A**) and after mixed meal intake (**B**) from patient (5 years post bariatric surgery). (**C-D**) Healthy control blood samples collected before (C) and after (D) mixed meal intake shows no hybridized bacterial cells. Instead, the auto-fluorescence of blood cells can be observed upon increased exposure. (**E-F**) Control of CARD-FISH efficiency on blood samples collected from the healthy control before (**E**) and after (**F**) mixed meal intake and deliberately infected with Pseudomonas putida (Red) prior to hybridization shows positively hybridized cells of P. putida (arrows) as expected. In blue, DAPI staining of blood cells is observed. Scale bar 10 µm for all images
